# Examination and diagnosis of electronic patient records and their associated ethics: a scoping literature review

**DOI:** 10.1186/s12910-020-00514-1

**Published:** 2020-08-24

**Authors:** Tim Jacquemard, Colin P. Doherty, Mary B. Fitzsimons

**Affiliations:** 1 FutureNeuro, the SFI Research Centre for Chronic and Rare Neurological Diseases, 123 Stephen’s Green, Dublin 2, Ireland; 2 Department of Neurology, St. James’s Hospital, James’s Street, Dublin 8, Ireland; 3 Trinity College Dublin, College Green, Dublin 2, Ireland

**Keywords:** Applied ethics, Healthcare, Electronic health records, Electronic patient records, eHealth, Review, Electronic medical records

## Abstract

**Background:**

Electronic patient record (EPR) technology is a key enabler for improvements to healthcare service and management. To ensure these improvements and the means to achieve them are socially and ethically desirable, careful consideration of the ethical implications of EPRs is indicated. The purpose of this scoping review was to map the literature related to the ethics of EPR technology. The literature review was conducted to catalogue the prevalent ethical terms, to describe the associated ethical challenges and opportunities, and to identify the actors involved. By doing so, it aimed to support the future development of ethics guidance in the EPR domain.

**Methods:**

To identify journal articles debating the ethics of EPRs, Scopus, Web of Science, and PubMed academic databases were queried and yielded 123 eligible articles. The following inclusion criteria were applied: articles need to be in the English language; present normative arguments and not solely empirical research; include an abstract for software analysis; and discuss EPR technology.

**Results:**

The medical specialty, type of information captured and stored in EPRs, their use and functionality varied widely across the included articles. Ethical terms extracted were categorised into clusters ‘privacy’, ‘autonomy’, ‘risk/benefit’, ‘human relationships’, and ‘responsibility’. The literature shows that EPR-related ethical concerns can have both positive and negative implications, and that a wide variety of actors with rights and/or responsibilities regarding the safe and ethical adoption of the technology are involved.

**Conclusions:**

While there is considerable consensus in the literature regarding EPR-related ethical principles, some of the associated challenges and opportunities remain underdiscussed. For example, much of the debate is presented in a manner more in keeping with a traditional model of healthcare and fails to take account of the multidimensional ensemble of factors at play in the EPR era and the consequent need to redefine/modify ethical norms to align with a digitally-enabled health service. Similarly, the academic discussion focuses predominantly on bioethical values. However, approaches from digital ethics may also be helpful to identify and deliberate about current and emerging EPR-related ethical concerns.

## Background

In 1971, Dr. Larry Weed, noted that good medical record keeping is intertwined with the delivery of high quality patient care [1, 2]. In this regard, electronic patient records (EPRs) facilitate better healthcare by providing timely access to comprehensive and organised patient information. Thus worldwide healthcare reform ambitions see EPR technology as a key enabler of improved population health, patient experience, value for money, and satisfaction and well-being of healthcare professionals (HCPs) [3, 4]. In addition, EPR technology or EPR-generated data combined with other digital technology, such as patient-facing interfaces providing patient access to their healthcare information [5], wearable devices to track patient’s daily routines [6], big-data analytics platforms based on linkage of population datasets [7], allow for an emerging generation of EPR associated benefits. To ensure the projected benefits and the means to achieve them are indeed socially and ethically desirable, careful consideration of the ethical implications of EPRs is indicated.

Previously reported challenges of EPRs in practice illustrate their ethical implications. For example, installation errors in imaging information technology systems can lead to erroneous health status reports with potential negative health outcomes [8]; poor attention to the required EPR related behavioural change management can add a disproportionate burden on HCPs time and negatively affect their work satisfaction [9]; EPR systems have been deployed with poor cybersecurity practices, which jeopardises the privacy and confidentiality of patient health data [10]; the sharing of patient data with commercial parties can damage patients’ trust in healthcare providers [11, 12]; and a failure to appreciate the limitations and biases in datasets can lead to the development of AI algorithms that unfairly privilege or discriminate against certain groups [13, 14]. In short, although EPR introduction is intended to benefit the quality, safety and efficiency of healthcare and health service delivery, it can also introduce unintended negative consequences. Therefore, to maximise desirable EPR impacts and minimise/eliminate the occurrence of adverse EPR sequalae, ethical concerns must be identified and addressed through all stages of the EPR system’s lifecycle from design through development, implementation and ongoing evolution.

Responsibility for safe, ethical and socially desirable application of EPRs in itself, requires a challenging apportionment across a complex network of actors [15]. Consider for example, where the locus of responsibility for patient privacy should lie. Is it with the: developer who uses encryption in the design of the EPR; healthcare system in the provision of secure data servers to host the EPR; clinicians/EPR users in choosing strong, and not sharing, passwords; patients who consent to sharing their information/waiving their privacy rights; policymakers for developing standards and regulations? Do these actors share all or some of the responsibility for patient privacy? How are the responsibility and accountability appropriately apportioned?

Since the advent of the first electronic repositories for recording and storing the health status of individuals in a clinical setting, a number of labels have been adopted to describe the ever-evolving capabilities of the technology [16–18]. While the electronic medical record (EMR) is confined to one healthcare practice [19], the electronic health record (EHR) contains a more complete record that is shareable between all providers involved in the individual’s healthcare [18, 20, 21]. Throughout this review, ‘EPR’ is used as an umbrella term to represent this full array of capabilities.

This paper reports a scoping literature review, which aimed to identify the prevalent ethical terms considered in the current literature in relation to the design, development and implementation of EPRs and to explore the associated opportunities, challenges and actors. Learning from this study may be used to inform future development of ethics guidance in the EPR domain. The review contributes to the existing academic discussion in four important ways. Firstly, it identifies terms with ethical connotations that are prevalent in the existent academic literature on EPR technology. Secondly, it explores the functionality, type of users, and actors baring rights and duties that influence EPR ethics (Additional file 1). Thirdly, it synthesizes the sources of evidence on the ethics of EPRs (Additional file 1). Fourthly, the review provides a critical analysis to identify gaps, shortcomings, and recurrent themes in this literature and can inform policy and practice regarding safe and ethical development, implementation, and use of EPR technology.

## Methods

This scoping review examined how EPR-related ethical values and principles are defined and understood; which actors are involved and their responsibilities; and what EPR functionalities and uses are discussed. In this regard, the review set out to explore the moral arguments about EPRs rather than to examine empirical research into people’s attitudes towards morality. For example, it considered the debate on importance of patient autonomy in relation to EPRs rather than the value patients place on the opportunity to have more control over their health data. The project team (Authors TJ, CD and MF) adopted the Preferred Reporting Items for Systematic reviews and Meta-Analyses extension for Scoping Reviews (PRISMA-ScR) checklist to guide the study [22] (see Additional file 2 for the checklist).

In contrast with a systematic review, which focuses on a narrow question with a predetermined study design [23, 24], the methodology of a scoping review allows for a broad scoping of the academic discussion [25, 26]. The utilised scoping review methodology is not outcome-based but rather aims to provide a critical analysis of the academic debate and its shortcomings [27]. Given the extensive debate on EPR ethics, the scoping literature helps to map the current state of affairs within the debate. Therefore, this type of review is appropriate when considering the research topic of EPR technology and its associated ethics.

### Information sources and search strategy

**Fig. 1:**
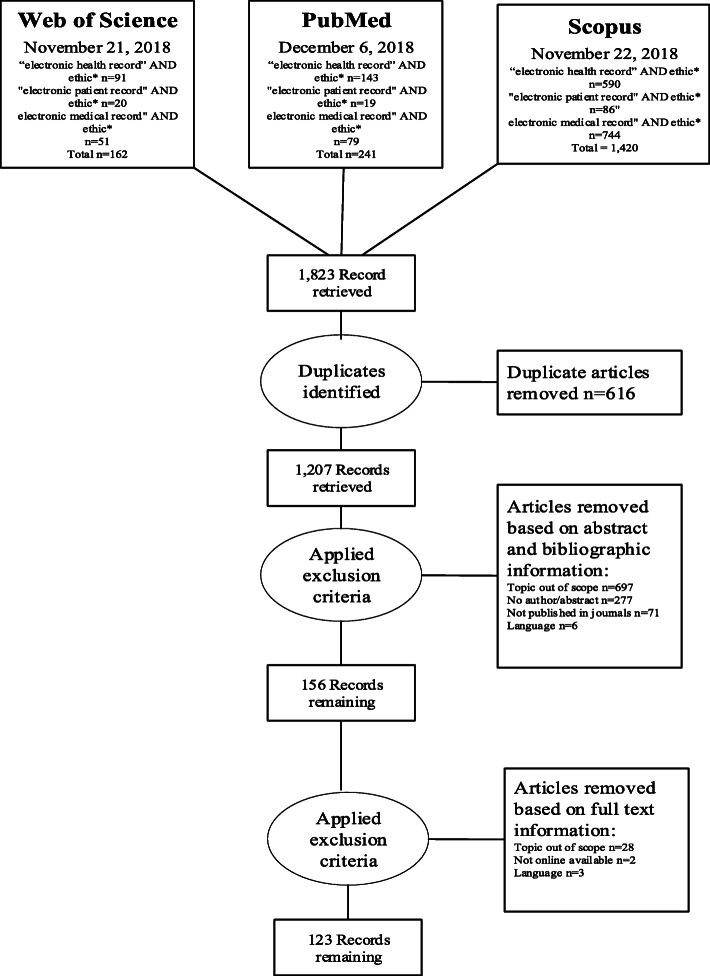
Overview of the article selection process

Three academic databases were queried: Scopus, Web of Science, and PubMed (Fig. 1). These were selected to ensure a minimum level of scientific validity while having both clinical and ethical relevance. Three search terms typically used to describe EPRs were used: (1) ‘electronic health record’, (2) ‘electronic patient record’, and (3) ‘electronic medical record’. These were combined with the term ‘ethic*’ to identify sources debating the ethics of EPRs. The wildcard, or the asterisk at the end of ‘ethic*’, allowed for variation, such as “ethics”, “ethical”, and “ethically”. Searches on Web of Science (November 21, 2018) and PubMed (December 6, 2018) databases were conducted without specifying data fields. The Scopus search (November 22, 2018) was limited to ‘article title’, ‘abstract’, and ‘keywords’ fields as without this limitation, Scopus searches examine the titles listed in the bibliography of articles, thus returning extraneous material.

### Inclusion and exclusion criteria

Eligibility conditions for the project were determined by the project team (TJ, CD, MF). Articles that met the following criteria were included:

- Journal articles:
  This criterion was applied to ensure a level of scientific validity.
- Those presenting normative ethical arguments regarding the design, development, implementation and use EPRs:
  The article had to be relevant to the purposes of this study.

Articles were excluded if they:

- Were not written in English:
  To ensure that all authors could read and judge the articles.
- Did not include an abstract:
  To facilitate the use of text analysis software to identify ethical terms used in the title and abstract of the articles. Also, the abstract allowed for a quick scan of the eligibility of the sources when the resources to conduct this research were limited.
- Simply mentioned the importance of ethical concerns or solely presented empirical research without a normative discussion or reasoning:
  The manuscript details the applications of moral considerations to EPR technology. In the absence of an ethical discussion, the article was judged irrelevant.
- Did not discuss EPR technology:
  To exclude articles irrelevant to the purposes of this study, which discusses EPR technology and its associated ethics.

### Search results and selection process

All stages of the selection process were reviewed at project team (TJ, CD, MF) meetings. A total of 1823 articles were identified for possible inclusion (Fig. 1). Following removal of duplicate citations, TJ assessed the eligibility of the remaining 1207 articles through reading their titles and abstracts. Then full texts of the remaining 156 articles were again examined by the same review author. This process yielded a final 123 eligible articles. To verify their eligibility, MF reviewed the titles and abstracts of the final 123 articles.

### Data extraction

A two-step approach was taken to the extraction and categorisation of data from the 123 eligible articles.

**Fig. 2:**
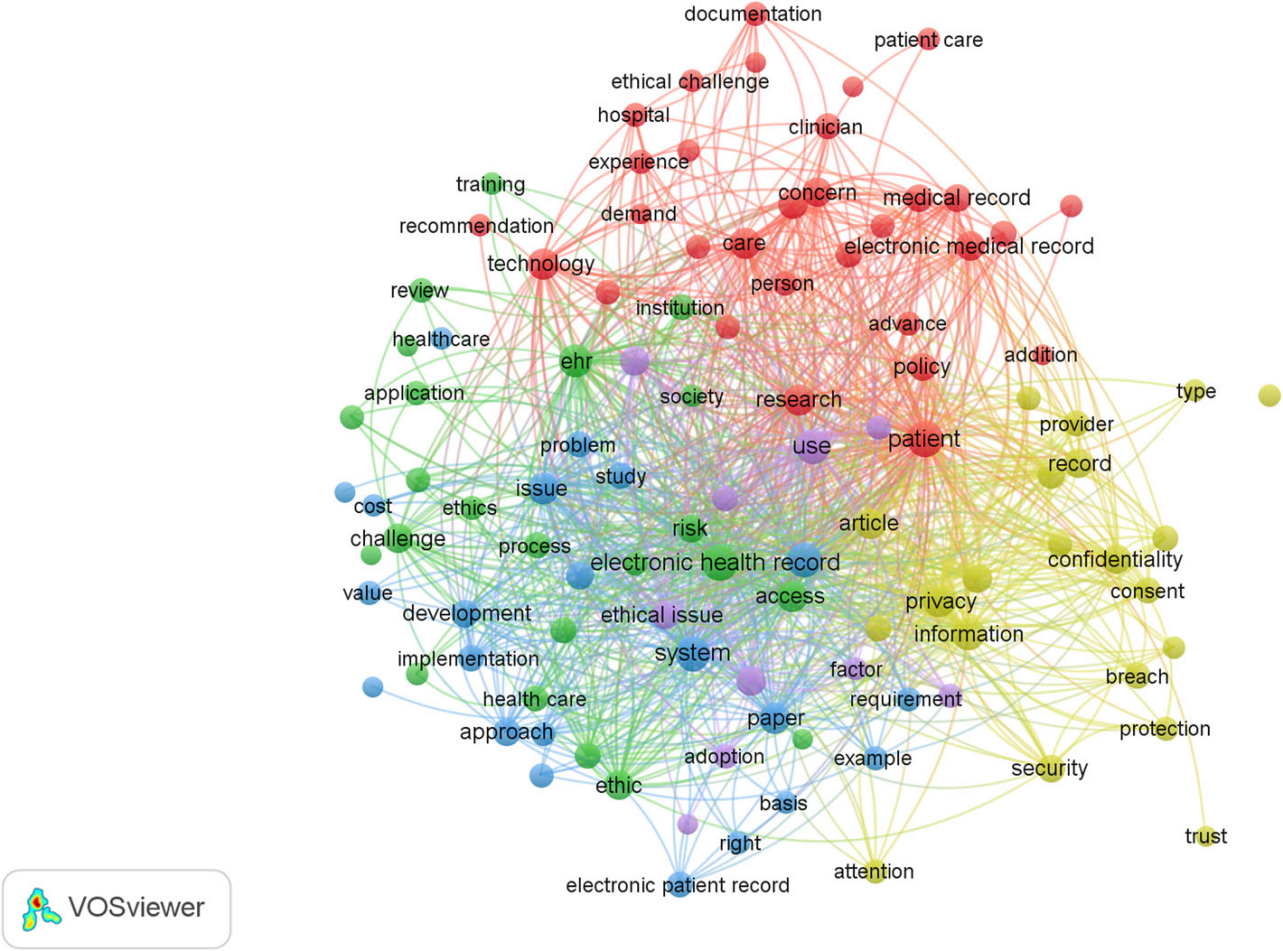
A visual representation of the full list of terms identified by VOSviewer

First, the titles and abstracts of the eligible articles were collated into a single CSV file. This file was then analysed using VOSviewer software [28] to identify terms (i.e. words/phrases that describe a concept or thing) and count the frequency of their occurrence with respect to the number of articles in which they appear. To cater for synonyms, VOSviewer applies a user-generated thesaurus file to merge terms (Additional file 3: Appendix C). For example, a variety of terms may be used to describe ‘clinician patient relationship’ (e.g. ‘physician patient relationship’; ‘doctor patient relationship’ etc). Those terms that were present in 6 or more of the 123 assessed articles were included in the VOSviewer count. A list of the resulting 113 VOSviewer generated terms is provided in Additional file 3: Appendix D and illustrated in Fig. 2.

From the list of 113 VOSviewer generated terms (Additional file 3: Appendix D), those indicative of ethical values, duties and rights were further manually extracted (column 2, Table 1) by TJ and MF. The resultant 16 terms guided a further re-reading of the full text of the 123 included articles by TJ to explore which ethical terms that were being considered, the functionality of the associated EPR, the users and uses of the technology, whose rights and duties are referred to, and which actors are seen as being responsible or accountable for protecting these rights (Additional file 1). A data charting Excel spreadsheet was jointly developed by TJ and MF to determine which variables to extract. During re-reading of the articles, TJ charted the data and further elucidated the meaning of the terms of interest, and even if they were ethically meaningful. For example, ‘benefits’ can indicate a material advantage, such as health insurance or sick pay [29], or an ethical concept indicating a good effect [30]. During this stage of the study, at repeated sessions, the project team (TJ, CD and MF) came together to discuss and critique the insights emerging from the review.

**Table 1:** Occurrence of ethical terms in the articles reviewed. Column 1: ranking based on frequency of occurrence. Column 2: the ethical term. Column 3: Number of included articles mentioning the term. Column 4: Cluster name

Ranking	Term	Occurrences	Cluster
1	privacy	41	Privacy
3	security	26	
2	confidentiality	28	
9	breach	12	
6	consent	21	Autonomy
10	control	11	
12	autonomy	9	
4	benefit	26	Risk/benefit analysis
5	risk	22	
7	quality	18	
11	safety	10	
14	efficiency	7	
8	clinician patient relationship	13	Human relationships
16	trust	6	
15	transparency	7	
13	responsibility	9	Responsibility

Finally, to aid the presentation of findings, following the full reading analysis each of the 16 ethical terms was assigned to one of 5 clusters (column 4, Table 1) which were defined and agreed by the project team (TJ, CD, MF). As clusters are not strictly defined, some terms could fit multiple categories. For example, the term ‘control’ is often associated with control over information sharing so could equally fit with either the ‘privacy’ or the ‘autonomy’ cluster.

## Results

The medical specialty, type of information captured and stored in EPRs, and their use and functionality varied widely across the articles included in this scoping review (Additional file 1). While most articles did not specify whether the EPR under consideration was confined to use within a single healthcare practice/organisation or was shared [30–52], others refer to challenges and opportunities related to sharing data across organisational boundaries [53–57], nationwide [58, 59] or even worldwide [21]. Some consider ethical issues that are associated with characteristics common to all EPR systems such as the nature of digital data [32, 60, 61], the confidentiality of health information [33, 39] or the use of the copy-paste functionality [38, 62, 63]. Others focus on ethical issues around a particular EPR use, such as health insurance claims [64], clinical governance [65], medical education [35, 66–69], health research [36, 70–73], predictive analytics [74], learning health system [41], genomics, biomarkers and photos [31, 34, 75–78], public health policies or surveillance [79–81], health service monitoring, evaluation and planning [82, 83]. The concept of providing patients access to their own medical record via electronic portals was of interest in many sources, particularly in relation to patients understanding the content of the record, provision of sufficient controls for patients to manage privacy, and patient responsibility for the accuracy of information in their healthcare record [31, 37, 84–92].

In the following, the opportunities, challenges and actors associated with EPR related ethical concerns debated in the literature are presented.

### Privacy

“Privacy”, and the related concepts of “confidentiality”, “breach” and “security” are grouped within a ‘Privacy’ cluster heading. Regarding EPR technology, privacy is a normative principle to evaluate arrangements around access to and distribution of personal, clinical information. As data sharing and exchange is integral to EPR systems, there is an unsurprising high frequency of occurrence of privacy-related ethical concerns (41 articles, which is 33% of the total). Privacy and confidentiality in medicine are closely related with the former often gained through the latter in the healthcare setting [70, 93]. Additionally, the terms ‘breach’ and ‘security’ were discussed in relation to confidentiality [39, 72, 94–98].

#### Challenges and opportunities

Most articles consider privacy a challenge to be overcome with privacy concerns sometimes seen as an obstacle to clinical EPR adoption [99] and to their use for purposes such as population health [100]. Third party usage of EPRs highlights particular privacy challenges [101], for example, when extracting data from EPRs for the purpose of: health insurance payments, research or sharing health information with employers regarding an individual’s fitness to work [83, 102–104]. Nevertheless, EPRs also offer opportunities regarding privacy as audit trails of date, time, and user information help monitor both appropriate and inappropriate access to the system [57, 105].

#### Actors

The sources invariably discussed the EPR-related privacy interests of patients and sometimes particularly vulnerable patient populations, such as children [106, 107] or in the mental health domain [108, 109]. Interestingly, even when discussing genomics, the privacy concerns of family members are not explicitly mentioned [31, 34, 60, 61, 75]. Although their use of the EPR could have implications for HCP privacy, this is not referred to in any of the sources reviewed. A variety of stakeholders are identified as having responsibility for EPR-related privacy. These include HCPs [110–113] in the clinical context or towards a patient following a data breach [95]; healthcare managers and policymakers [21, 41, 45, 70, 97, 99, 109, 114, 115]; and information technology (IT) specialists [116]. Some articles acknowledge that privacy is a shared responsibility [37, 96, 117–120]. Many sources mention that privacy challenges should be addressed through technological design [56, 84, 86, 89, 121, 122].

### Autonomy

Articles that considered any of the related concepts of ‘autonomy’, ‘consent’, and ‘control’ were grouped under the Autonomy cluster heading. While autonomy is the ability of an individual to self-govern, consent is an instrument that allows people to exercise this right. ‘The term ‘control’ was regularly discussed regarding the patient’s right to determine what happens with their EPR-based personal information [43, 56, 105, 123, 124].

#### Challenges and opportunities

EPR technology can promote patient autonomy. Portals to the EPR provide patients with access to their clinical data and enables greater patient participation in decision-making around their healthcare [88, 123, 125, 126]. Only a few articles advocate a requirement for patient consent when the EPR is being used for clinical purposes [45, 109, 127]. However, consent and the type of consent appears more critical when using EPR-based information outside the clinical doctor-patient relationship [46, 108]. Such secondary uses include research [31, 71–73, 80, 128], and clinical training when medical students track patients to learn about their on-going treatment and outcomes [66].

Furthermore, consent is indicated when sensitive data types such as genomic data [31, 34], photographic images, [78] or biobank data [77], are involved. Likewise, where children [106] and adolescents whose decision-making competence is evolving [49, 118], or patients with mental health issues [109, 113, 129] are concerned, issues around consent and EPRs are more complex. A need for patient awareness about how algorithms within EPRs function was also highlighted [129, 130].

#### Actors

While patient autonomy is mainly considered, some articles also refer to the intellectual and clinical freedom of HCPs who record clinical care using the EPR [126, 131]. Clinicians’ documentation may be constrained as they fear patients with access to their own EPR may read and potentially misinterpret sensitive information regarding their physical and mental well-being [126]. The potential for increased scrutiny of individual care practitioners whose work and actions can be more visible with EPR technology [131] was also mentioned.

### Risk/benefit

The terms ‘Benefit’, ‘Risks’, ‘Safety’, ‘Quality’ and ‘Efficiency’, related to preventing harm and realising benefits, were clustered under the heading Risk/benefit. Ethical considerations required a favourable benefits/risks balance of EPRs [30, 76, 132–136] and many saw this balance as a challenge [30, 58, 80, 94, 123, 136–138]. Some discussed EPR technology in terms of harm to the patient [30, 39, 62, 64, 66, 80, 118, 123, 131, 137] or mentioned patient safety [57, 122, 138]. Concepts of ‘quality’ and ‘efficiency’, instruments to obtaining the benefits from EPR systems, were also explored [65, 122, 139, 140].

#### Challenges and opportunities

Patients, clinicians and the wider healthcare system can profit from the use of EPRs by better quality and continuity of care, improved health outcomes and avoidance of medical error [88, 94, 122, 131, 137, 141]. EPR-derived benefits are often to the larger community or other parties and not directly to individual patients or clinicians [128]. Examples include health service performance management and predictive analytics [80], capitalising on the commercial value of health data [142] and population health surveillance [79]. Managing the tension between the individual patients’ right to autonomy and the public good [105] that can be derived from EPR-supported medical education [68, 143] and scientific research presents a significant challenge [67, 71, 135, 142].

Of particular concern is the burden on clinicians’ time, who, for instance, have to invest time in using the EPR or answering patients who seek clarification as a result of patient access to EPR data [43, 126, 138]. Additionally, EPR benefits may be unequally distributed between patients groups [47, 83, 90]. For example, the development of EPRs for paediatric healthcare can be complicated [144], as can the digital capture of information in the mental health domain [145].

#### Actors

Duties to maximise benefits and minimise risks are assigned to a variety of stakeholders. HCPs must avoid EPR data inaccuracies through safe and responsible use of the system [38, 63]. Researchers who use the EPR, software developers, EPR administrators, and healthcare policymakers each have responsibility for EPR reliability so that benefits can be reaped [141]. Individual patients also have obligations, as use of their data can create a common/societal benefit by informing science and continuous quality improvement in healthcare [41, 42, 128, 135, 136].

### Human relationships

Human relationships are important as a source of intimacy, social wellbeing, and human dignity [146]. As determinants of human relationships, trust [73, 114, 127, 147] and transparency [53, 147] are grouped with clinician-patient relationship under this cluster heading.

#### Challenges and opportunities

EPRs can enhance trust between patients and healthcare professionals, as the ease of sharing digital information helps communication [53, 147]. Likewise, when patients have access to their own healthcare record through electronic portals, the information gap between them and their clinician is reduced and a more balanced patient–clinician relationship ensues [88, 115].

However, patient portal functionality may cause HCPs to purposely begin obfuscating clinical information, such as impressions of mental well-being, making it more difficult for patients to understand their healthcare record and thus hampering the relationship between clinicians and patients [126]. Clinical data sharing between HCPs and organisations enabled by the EPR can also cause patients’ reluctance to disclose relevant information, as they perceive a potential for infringement of their confidentiality [52, 82, 106, 127, 141, 148]; for example because of inappropriate or unauthorised access to healthcare records [147] or from unsupported use of their EPR data for purposes such as research or public health [42, 53, 64].

In addition, EPR technology may leave less room for human interaction and interpretation. The standardised structure of EPRs may limit documentation of individual patient nuances and narrow information captured by HCPs about their patients [32, 149–152]. As a result, the patient may be seen as a series of data points rather than a human [82, 130, 153, 154]. Furthermore, the EPR computer is sometimes seen as a third party that distracts from the intimacy of the doctor-patient relationship [51, 63, 83, 149].

#### Actors

The articles almost exclusively discuss the relationship between patient and healthcare provider [43, 53, 82, 83, 87, 94, 127, 138, 148, 149, 155]. In contrast, duties to safeguard this relationship and promote trust and transparency is distributed among a range of stakeholders and processes: designers or developers of the EPR technology [60, 126], institutional oversight [53, 147], educational institutions [155], organisational processes [82, 126], healthcare providers [43, 83, 94, 127, 141, 148, 149], policymakers [21, 44, 87, 142], healthcare management [54] and dynamic consent models that improve patients’ trust in how data is used [73].

### Responsibility

Responsibility is interpreted as the allocation of duties and obligations as a result of the design, implementation, and use of EPR systems. As the analysis of articles included in this review did not expose any other relevant values (e.g. accountability) for this cluster heading only the single ‘responsibility’ value is represented.

#### Challenges and opportunities

Implementation of EPRs as well as patient portal functionality disrupts conventional processes and creates new responsibilities for moral issues such as confidentiality [49, 95, 115, 141], informed patient consent [156], data accuracy or clinical decision support systems [57, 87, 145]. Electronic portals to EPRs provide patients and/or their legal guardians with an opportunity to take more control of, and responsibility for, the management of their healthcare data and healthcare [85, 87, 92]. However, when patients obtain access to their medical record, issues around data custodianship and apportioning responsibility become increasingly challenging [84].

#### Actors

Responsibility for the safe and ethical adoption of EPR technology spans multiple stakeholders [87]. The roles and responsibilities of healthcare professionals, healthcare organisations and healthcare policymakers for responsible use of EPRs and for maintaining best practices are debated in many articles [31, 38, 43, 49, 54, 57, 83, 85, 94, 95, 111, 139, 141, 145, 157]. Information technology (IT) personnel also have responsibilities for example, for securing the required infrastructure [121] or an ethical development of technology [130, 158, 159].

## Discussion

In this scoping literature review, the wide spectrum of EPR-related ethical and social issues debated in the academic literature is distilled into one manuscript. Four important lessons that can inform the design, development and implementation of EPRs emerge from this review. First, the purpose for which an EPR system is used affects the ethical assessment. Application of EPRs to support patient care for clinical purposes seems less ethically challenging compared to EPR use to facilitate medical training and education, for research and managerial purposes, or when EPR-based data is shared with other non-clinical stakeholders (e.g. health insurance claims). Second, ethical concerns will be influenced by the data subject population (e.g. children; the mental health domain) and the type/sensitivity of data/information (e.g. genomic data) stored within the EPR. Third, EPRs involve a wide variety of stakeholders with rights and/or responsibilities regarding the safe and ethical use of the technology. For example, EPR ethics can privilege the rights of the patient, however, the patient may have concurrent duties to promote a public good, such as better quality and safety of healthcare, through research based on their healthcare data. Fourth, there is a strong consensus within the literature on the importance and relevance of separate ethical terms (Table 1) discussed in relation to EPRs. None of the articles argued that an ethical term mentioned in the academic discussion on the ethics of EPRs was irrelevant or misguided.

The introduction of EPRs is a multidimensional disruption intended to benefit healthcare and health service delivery. However, they can also bring unintended negative consequences. An ethical analysis is therefore critical to assuring quality and managing risk associated with EPR interventions. Although the current academic literature is informative in terms of identifying EPR-related ethical considerations and their determinants, at times it lacks analytic depth and fails to take account of the need to redefine/modify ethical norms to align with a digitally enabled health service. For example, privacy is generally discussed in a manner more in keeping with a traditional model of healthcare delivery rather than taking account of the multidimensional ensemble of factors at play in the EPR era. Similarly regarding autonomy, the role the technology plays in shifting from a traditional medical paternalism to more mutual partnerships between HCPs and patients indicates deeper examination of EPR implications for patient autonomy.

Our interests in privacy are mediated by moral, social and legal norms, which are affected and altered through technology [160]. EPRs facilitate the sharing and exchange of patient information across organisational boundaries (e.g. within and between different healthcare settings) thereby allowing delivery of healthcare to be more integrated between teams of HCPs. However, privacy discussion in the current literature on EPR-related ethics is generally limited to views on the patient’s right to control who has access to their health data and the traditional concept of clinician-patient confidentiality. Future research should inform the definition of privacy norms that are more reflective of the relevance of EPR-enabled integrated healthcare teams and the relationships mediating patient care.

Respect for autonomy can broadly be understood to include rights to form one’s own values and beliefs, and to act in accordance with them. As EPRs can empower patients by giving them access to their own healthcare record, they may form beliefs about their care and act upon them. Although the implications are considerably far-reaching, the current literature on EPR-related autonomy is more closely related to privacy as it mainly focuses on patient decisions around disclosure and use of their data. The ways in which EPRs can strengthen patient autonomy through, for example, shared decision-making warrants a more in-depth examination of the full spectrum of autonomy in order to improve our ethical understanding of the technology.

### Limitations

The potential for bias in this study is acknowledged. For example, one author (TJ), assessed the eligibility of articles and analysed the full text of those included. To reduce the possibility of bias resulting from TJ’s interpretation, MF verified the eligibility of the final 123 included articles by reviewing their titles and abstracts and oversight of all stages of the process involved the full project team (TJ, CD, MF) who agreed the project design, database search strings, inclusion and exclusion criteria, and discussed and critiqued the insights emerging from the review. Our pragmatic approach was taken due to limited resource availability.

While the three databases searched for the purposes of this scoping literature review cover much of the relevant academic literature, it is possible that not all of the EPR-related ethical debate has been comprehensively captured. As only peer-reviewed scientific literature was included, the potential to include relevant debate from other sources was dismissed. Similarly, the interval between the database searches in November and December 2018 and the resultant manuscript may also be seen as a limitation. However, such intervals are occasionally seen in published scoping reviews as time is required to study the identified articles, to interpret and assess the extracted data, and to construct a balanced narrative of the subject [161]. Moreover, rather than being the final word on the topic, our goal in conducting this review was to promote greater awareness of applied ethics in the EPR domain and encourage other researchers to deliberate on them.

In order to address the most frequently occurring ethical values in the literature, at the VOSviewer software-enabled text analysis step, a threshold was set to include those that occurred in 6 or more articles. It is possible that this threshold resulted in some EPR-related ethical terms being missed. For simplicity, the ethical principles that were exposed in this review were clustered under 5 headings and may have limited presentation of certain intricacies of the ethical arguments.

Notwithstanding these limitations, this review provides a foundation for future EPR-related ethics research and the development of a framework to guide safe and ethical implementation, development, and use of EPR technology. As a protocol for our study is not publicly shared, further information about its design is available on request from the corresponding author.

### Future challenges

Apart from one article reporting the first steps in establishing a framework using a privacy and ethics consensus development process [30], no fully developed EPR ethics framework was uncovered during this scoping review. An ethical framework formulated to inform the design, development, implementation and use of EPR systems would aid healthcare organisations and should describe the roles and responsibilities of diverse stakeholders.

Although significant EPR technological developments, such as artificial intelligence, natural speech analysis and integration with wearable devices, are expected, only a few of the articles reviewed discuss these advances [161–163]. To reap the next generation of benefits, values associated more closely with digital technology ethics and digitisation in the wider societal context require more consideration in, and could help inform, the EPR arena. The current academic discussion on EPRs focuses primarily on biomedical issues. However, both biomedical and digital technology ethics, specialisations within the field of applied ethics, offer approaches to identify and deliberate about EPR-related ethical concerns. Ethics in the biomedical domain, for example, can be utilised to evaluate norms to govern the disclosure of information required for informed consent [164]. Meanwhile, digital technology ethics can be used for appraisal of issues such as the degree of control users should have regarding the functioning of algorithms embedded in the technology [165] and can help elucidate issues around market power, monopolisation, vendor lock-in, bias and transparency of algorithms [166]. Furthermore, in a healthcare system in which care is increasingly mediated by EPR technology, the relationship between varieties of stakeholders should be considered. Nevertheless, the literature mainly focuses on the impact of EPR technology on the clinician-patient relationship. Future ethical assessments should reflect the importance of the relationship between IT specialists or system vendors and HCPs or healthcare managers.

## Conclusions

Internationally, healthcare reform policies promote adoption of EPR technology to create conditions for better quality, safety and value of services. While EPRs have existed for several decades, their functionality, utility and adoption are ever-evolving. This review presents the array of determinants of the ethical and moral questions to be addressed in order to safely unlock the opportunities presented by this maturing and dynamic technology. To reap the next generation of benefits from EPRs, an ensemble of stakeholders and ethical values and their associated challenges and opportunities should be considered across the EPR life-cycle from concept, through design, development and implementation, and on to sustained operation of the system. Without such careful attention, EPRs may be utilised for a variety of practical goals that conflict with the fiduciary duty of care towards the patient, and may diminish trust in this powerful technology.

## Supplementary information

**Additional file 1.** Overview of sources.

**Additional file 2.** Preferred Reporting Items for Systematic reviews and Meta-Analyses extension for Scoping Reviews (PRISMA-ScR) Checklist.

**Additional file 3.** Appendix C: Thesaurus file. Appendix D: Overview of terms.

## Data Availability

Not applicable.

## Abbreviations

EHR: Electronic health record
EMR: Electronic medical record
EPR: Electronic Patient Record
HCP: Healthcare Professional
IT: Information technology

## References

[1] Aronson MD (2019). The purpose of the medical record: why Lawrence weed still matters. Am J Med.

[2] VisualDx. Larry Weed’s 1971 Internal Medicine Grand Rounds. YouTube. 2012.

[3] Bodenheimer T, Sinsky C (2014). From triple to quadruple aim: care of the patient requires care of the provider. Ann Fam Med.

[4] Sikka R, Morath JM, Leape L (2015). The quadruple aim: care, health, cost and meaning in work. BMJ quality &amp. Safety..

[5] Dendere R, Slade C, Burton-Jones A, Sullivan C, Staib A, Janda M (2019). Patient portals facilitating engagement with inpatient electronic medical records: a systematic review. J Med Internet Res.

[6] Dinh-Le C, Chuang R, Chokshi S, Mann D. Wearable Health Technology and Electronic Health Record Integration: Scoping Review and Future Directions. JMIR Mhealth Uhealth. 2019;7(9):e12861-e. 10.2196/12861 PMC6746089 31512582

[7] Combi C, Pozzi G (2019). Clinical information systems and artificial intelligence: recent research trends. Yearb Med Inform.

[8] Cullen P. International alert issued after HSE computer glitch. Irish Times 2017.

[9] Gawande A. Why doctors hate their computers the new Yorker. 2018.

[10] Grauer Y. Why is the healthcare industry still so bad at cybersecurity? Ars Technica: Conde Nast; 2020 [Available from: https://arstechnica.com/information-technology/2020/02/why-is-the-healthcare-industry-still-so-bad-at-cybersecurity/.

[11] Helm T (2020). Revealed: how drugs giants can access your health records. Guardian.

[12] Evans M. Hospitals give tech giants access to detailed medical records. Wall Street J. 2020.

[13] Ross C, Swetlitz I. IBM pitched its Watson supercomputer as a revolution in cancer care. It’s nowhere close. Stat News. 2017 5 September 2017.

[14] Ledford H. Millions of black people affected by racial bias in health-care algorithms. Nature. Nature. 2019;574:608–9. 10.1038/d41586-019-03228-6. 10.1038/d41586-019-03228-6 31664201

[15] Van den Hoven J. Value sensitive design and responsible innovation. Responsible Innovation: Managing the Responsible Emergence of Science and Innovation in Society. 2013. p. 75–83.

[16] ISO. Business requirements for health summary records — Part 1: Requirements Online Browsing Platform (OBP): ISO; 2009 [ISO/TR 12773–1:2009(en):[Available from: https://www.iso.org/obp/ui/#iso:std:iso:tr:12773:-1:ed-1:v1:en.

[17] Greenhalgh T, Potts HWW, Wong G, Bark P, Swinglehurst D (2009). Tensions and paradoxes in electronic patient record research: a systematic literature review using the meta-narrative method. Milbank Q.

[18] Office of the CIO. National Electronic Health Record. Health Service Executive; 2015. Contract No.: Version 1.

[19] McMullen PC, Howie WO, Philipsen N, Bryant VC, Setlow PD, Calhoun M (2014). Electronic Medical Records and Electronic Health Records: Overview for Nurse Practitioners. J Nurse Pract.

[20] Gunter TD, Terry NP. The emergence of national electronic health record architectures in the United States and Australia: models, costs, and questions. J Med Internet Res. 2005;7(1):e3-e. 10.2196/jmir.7.1.e3 PMC1550638 15829475

[21] Scott RE, Jennett P, Yeo M (2004). Access and authorisation in a Glocal e-health policy context. Int J Med Inform.

[22] Tricco AC, Lillie E, Zarin W, O'Brien KK, Colquhoun H, Levac D (2018). PRISMA extension for scoping reviews (PRISMA-ScR): checklist and explanation. Ann Intern Med.

[23] Munn Z, Peters MDJ, Stern C, Tufanaru C, McArthur A, Aromataris E (2018). Systematic review or scoping review? Guidance for authors when choosing between a systematic or scoping review approach. BMC Med Res Methodol.

[24] Pham MT, Rajić A, Greig JD, Sargeant JM, Papadopoulos A, McEwen SA (2014). A scoping review of scoping reviews: advancing the approach and enhancing the consistency. Res Synth Methods.

[25] Levac D, Colquhoun H, O'Brien KK (2010). Scoping studies: advancing the methodology. Implement Sci.

[26] Arksey H, O’Malley L (2005). Scoping studies: towards a methodological framework. Int J Soc Res Methodol.

[27] Grant MJ, Booth A (2009). A typology of reviews: an analysis of 14 review types and associated methodologies. Health Inform Libraries J.

[28] Centre for Science and Technology Studies. Welcome to VOSviewer The Netherlands: Leiden University; 2018 [Available from: https://www.vosviewer.com/.

[29] Rothstein MA, Talbott MK (2007). Compelled authorizations for disclosure of health records: magnitude and implications. Am J Bioeth.

[30] Liyanage H, Liaw ST, Di Iorio CT, Kuziemsky C, Schreiber R, Terry AL (2016). Building a privacy, ethics, and data access framework for real world computerised medical record system data: a Delphi study Contribution of the Primary Health Care Informatics Working Group. Yearb Med Inform.

[31] Nishimura AA, Tarczy-Hornoch P, Shirts BH (2014). Pragmatic and ethical challenges of incorporating the genome into the electronic medical record. Curr Genet Med Rep.

[32] Roberts A (2017). Language, structure, and reuse in the electronic health record. AMA J Ethics.

[33] Accordino R, Kopple-Perry N, Gligorov N, Krieger S (2014). The medical record as legal document: when can the patient dictate the content? An ethics case from the Department of Neurology. Clin Ethics.

[34] Al Mallah A, Guelpa P, Marsh S, van Rooij T (2010). Integrating genomic-based clinical decision support into electronic health records. Personalized Med.

[35] Altman M (2007). The clinical data repository: a challenge to medical student education. J Am Med Inform Assoc.

[36] Hollister B, Bonham VL (2018). Should electronic health record-derived social and behavioral data be used in precision medicine research?. AMA J Ethics.

[37] Bakker A (2004). Digest of the discussion group sessions. Realising security of the electronic record. Int J Med Inform.

[38] Bernat JL (2013). Ethical and quality pitfalls in electronic health records. Neurology..

[39] Bhuyan SS, Bailey-DeLeeuw S, Wyant DK, Chang CF. Too Much or Too Little? How Much Control Should Patients Have Over EHR Data? J Med Syst. 2016;40(7). 10.1007/s10916-016-0533-2 27272134

[40] Casanovas P, Mendelson D, Poblet M (2017). A linked democracy approach for regulating public health data. Heal Technol.

[41] Friedman C, Rigby M (2013). Conceptualising and creating a global learning health system. Int J Med Inform.

[42] Goodman KW (2010). Ethics, information technology, and public health: new challenges for the clinician-patient relationship. J Law Med Ethics..

[43] Haig SV (2010). Ethical choice in the medical applications of information theory. Clin Orthop Relat Res.

[44] Huser V, Cimino JJ (2014). Don't take your EHR to heaven, donate it to science: legal and research policies for EHR post mortem. J Am Med Inform Assoc.

[45] Kluge EH (2004). Informed consent and the security of the electronic health record (EHR): some policy considerations. Int J Med Inform.

[46] Kluge EHW. Informed consent to the secondary use of EHRs: Informatic rights and their limitations. Stud Health Technol Inform. 2004:635–8. 15360890

[47] Layman EJ (2008). Ethical issues and the electronic health record. Health Care Manager.

[48] McBride S, Tietze M, Robichaux C, Stokes L, Weber E. Identifying and addressing ethical issues with use of electronic health records. Online J Issues Nurs. 2018;23(1).

[49] Nielsen BA, Baum RA, Soares NS (2013). Navigating ethical issues with electronic health records in developmental-behavioral pediatric practice. J Dev Behav Pediatr.

[50] Nielsen BA (2015). Confidentiality and electronic health records: keeping up with advances in technology and expectations for access. Clin Pract Pediatr Psychol.

[51] Strain J, Botin L (2007). A phenomenological perspective on clinical communication and interaction: the case of electronic health records. J Inf Commun Ethics Soc.

[52] Wangenheim PM (2018). Scribes, electronic health records, and the expectation of confidentiality. J Clin Ethics..

[53] Francis LP (2010). The physician-patient relationship and a national health information network. J Law Med Ethics..

[54] Milton CL (2009). Information sharing: transparency, nursing ethics, and practice implications with electronic medical records. Nurs Sci Q.

[55] Iacovino L, Reed B (2008). Recordkeeping research tools in a multi-disciplinary context for cross-jurisdictional health records systems. Arch Sci.

[56] Wainer J, Campos CJ, Salinas MD, Sigulem D (2008). Security requirements for a lifelong electronic health record system: an opinion. Open Med Inform J.

[57] Sittig DF, Singh H (2011). Legal, ethical, and financial dilemmas in electronic health record adoption and use. Pediatrics..

[58] Phillips W, Fleming DA (2010). Moral and prudential considerations in adopting electronic medical records. Mo Med.

[59] Pirnejad H, Bal R, Stoop AP, Berg M (2007). Inter-organisational communication networks in healthcare: centralised versus decentralised approaches. Int J Integrated Care.

[60] McGuire AL, Fisher R, Cusenza P, Hudson K, Rothstein MA, McGraw D, Matteson S, Glaser J, Henley DE (2008). Confidentiality, privacy, and security of genetic and genomic test information in electronic health records: points to consider. Genet Med.

[61] McGuire AL, Basford M, Dressler LG, Fullerton SM, Koenig BA, Li R, McCarty CA, Ramos E, Smith ME, Somkin CP, Waudby C, Wolf WA, Clayton EW (2011). Ethical and practical challenges of sharing data from genome-wide association studies: the eMERGE consortium experience. Genome Res.

[62] Weis JM, Levy PC (2014). Copy, paste, and cloned notes in electronic health records;prevalence, benefi ts, risks, and best practice recommendations. Chest..

[63] Phillips W, Fleming D (2009). Ethical concerns in the use of electronic medical records. Mo Med.

[64] Lercher A (2008). A social contract for health information. J Inform Ethics.

[65] Sanelli-Russo S, Folkers KM, Sakolsky W, Fins JJ, Dubler NN (2018). Meaningful use of electronic health Records for Quality Assessment and Review of clinical ethics consultation. J Clin Ethics.

[66] Brisson GE, Barnard C, Tyler PD, Liebovitz DM, Neely KJA (2018). framework for tracking former patients in the electronic health record using an educational registry. J Gen Intern Med.

[67] Brisson GE, Neely KJ, Tyler PD, Barnard C (2015). Privacy versus confidentiality: more on the use of the electronic health record for learning. Acad Med.

[68] McLaughlin K, Coderre S (2015). Finding the middle path in tracking former patients in the electronic health record for the purpose of learning. Acad Med.

[69] Okada M, Yamamoto K, Watanabe K (2007). Conceptual model of health information ethics as a basis for computer-based instructions for electronic patient record systems. Stud Health Technol Inform.

[70] Anuradha C, Babu PBR (2012). Securing privacy for confidential databases using anonymization. Middle East J Sci Res.

[71] Lowrance WW (2003). Learning from experience: privacy and the secondary use of data in Health Research. J Biolaw Bus.

[72] Spector-Bagdady K, Shuman AG (2018). Reg-ent within the learning health system. Otolaryngol Head Neck Surg.

[73] Williams H, Spencer K, Sanders C, Lund D, Whitley EA, Kaye J, Dixon WG (2015). Dynamic consent: a possible solution to improve patient confidence and trust in how electronic patient records are used in medical research. JMIR Med Inform.

[74] Amarasingham R, Audet AM, Bates DW, Glenn Cohen I, Entwistle M, Escobar GJ, Liu V, Etheredge L, Lo B, Ohno-Machado L, Ram S, Saria S, Schilling LM, Shahi A, Stewart WF, Steyerberg EW, Xie B (2016). Consensus Statement on Electronic Health Predictive Analytics: A Guiding Framework to Address Challenges. EGEMS (Washington, DC).

[75] Hazin R, Brothers KB, Malin BA, Koenig BA, Sanderson SC, Rothstein MA, Williams MS, Clayton EW, Kullo IJ (2013). Ethical, legal, and social implications of incorporating genomic information into electronic health records. Genet Med.

[76] Shoenbill K, Fost N, Tachinardi U, Mendonca EA (2014). Genetic data and electronic health records: a discussion of ethical, logistical and technological considerations. J Am Med Inform Assoc.

[77] Caenazzo L, Tozzo P, Borovecki A (2015). Ethical governance in biobanks linked to electronic health records. Eur Rev Med Pharmacol Sci.

[78] Lakdawala N, Fontanella D, Grant-Kels JM (2012). Ethical considerations in dermatologic photography. Clin Dermatol.

[79] Hoffman S, Podgurski A (2013). Big bad data: law, public health, and biomedical databases. J Law Med Ethics.

[80] Cato KD, Bockting W, Larson E (2016). Did I tell you that? Ethical issues related to using computational methods to discover non-disclosed patient characteristics. J Empirical Res Hum Res.

[81] Eggleston EM, Weitzman ER. Innovative uses of electronic health records and social media for public health surveillance. Curr Diab Rep. 2014;14(3). 10.1007/s11892-013-0468-7 24488369

[82] de Ruiter HP, Liaschenko J, Angus J (2016). Problems with the electronic health record. Nurs Phil.

[83] Sulmasy LS, Lopez AM, Horwitch CA (2017). Ethical implications of the electronic health record: in the service of the patient. J Gen Intern Med.

[84] Beard L, Schein R, Morra D, Wilson K, Keelan J (2012). The challenges in making electronic health records accessible to patients. J Am Med Inform Assoc.

[85] Davis KA, Smith LB (2016). Ethical considerations about EHR-mediated results disclosure and pathology information presented via patient portals. AMA J Ethics.

[86] Furano RF, Kushniruk A, Barnett J (2017). Deriving a set of privacy specific heuristics for the assessment of PHRs (personal health records). Stud Health Technol Inform.

[87] Garrety K, McLoughlin I, Wilson R, Zelle G, Martin M (2014). National electronic health records and the digital disruption of moral orders. Soc Sci Med (1982).

[88] Lee CI, Langlotz CP, Elmore JG (2016). Implications of direct patient online access to radiology reports through patient web portals. J Am Coll Radiol.

[89] Quantin C, Coatrieux G, Allaert FA, Fassa M, Bourquard K, Boire JY (2009). New advanced technologies to provide decentralised and secure access to medical records: case studies in oncology. Cancer Informat.

[90] Spriggs M, Arnold MV, Pearce CM, Fry C (2012). Ethical questions must be considered for electronic health records. J Med Ethics.

[91] Tussey CM, Marcopulos BA, Bush SS (2015). Evolving roles, innovative practice, and rapid technology growth: remaining ethical in modern clinical neuropsychology. Psychol Injury Law.

[92] Wynia M, Dunn K (2010). Dreams and nightmares: practical and ethical issues for patients and physicians using personal health records. J Law Med Ethics..

[93] Allen AL. Privacy and confidentiality in medicine are closely related. In: Zalta EN, editor. The Stanford Encyclopedia of Philosophy. Winter 2016 Edition ed2015.

[94] Lo B (2006). Professionalism in the age of computerised medical records. Singap Med J.

[95] Kim D, Schleiter K, Crigger BJ, McMahon JW, Benjamin RM, Douglas SP (2010). A physician’s role following a breach of electronic health information. J Clin Ethics.

[96] Neame RLB (2014). Privacy protection for personal health information and shared care records. Inform Prim Care.

[97] Sade RM (2010). Breaches of health information: are electronic records different from paper records?. J Clin Ethics..

[98] Satkoske VB, Parker LS (2010). Practicing preventive ethics, protecting patients: challenges of the electronic health record. J Clin Ethics..

[99] Ben-Assuli O (2015). Electronic health records, adoption, quality of care, legal and privacy issues and their implementation in emergency departments. Health Policy (Amsterdam, Netherlands).

[100] Jensen PB, Jensen LJ, Brunak S (2012). Mining electronic health records: towards better research applications and clinical care. Nat Rev Genet.

[101] McSherry B (2004). Third party access to shared electronic mental health records: ethical issues. Psychiatry Psychol Law.

[102] Veronesi JF (1999). Ethical issues in computerized medical records. Crit Care Nurs Quart.

[103] Wilburn A (2018). Nursing informatics: ethical considerations for adopting electronic records. NASN School Nurse (Print).

[104] Balka E, Tolar M (2011). Everyday ethical dilemmas arising with electronic record use in primary care. Stud Health Technol Inform.

[105] Fry CL, Spriggs M, Arnold M, Pearce C (2014). Unresolved ethical challenges for the Australian personally controlled electronic health record (PCEHR) system: key informant interview findings. AJOB Empirical Bioethics.

[106] Slabbert MN (2005). Parental access to minors' health records in the south African health care context: concerns and recommendations. Med Law.

[107] Smolyansky BH, Stark LJ, Pendley JS, Robins PM, Price K (2013). Confidentiality and electronic medical records for behavioral health records: the experience of pediatric psychologists at four children's hospitals. Clin Pract Pediatr Psychol.

[108] McSherry B (2004). Ethical issues in HealthConnect’s shared electronic health record system. J Law Med.

[109] Clemens NA (2012). Privacy, consent, and the electronic mental health record: the person vs. the system. J Psychiatr Pract.

[110] Wallace IM (2015). Is patient confidentiality compromised with the electronic health record?: a position paper. CIN.

[111] Kluge EHW (1998). Fostering a security culture: a model code of ethics for health information professionals. Int J Med Inform.

[112] Barber A (2012). Computers for physicians: never do harm. Care Manag J.

[113] Ashton K, Sullivan A (2018). Ethics and confidentiality for psychologists in academic health centers. J Clin Psychol Med Settings.

[114] McGuire AL, Fisher R, Cusenza P, Hudson K, Rothstein MA, McGraw D (2008). Confidentiality, privacy, and security of genetic and genomic test information in electronic health records: points to consider. Genet Med.

[115] Stahl BC, Doherty NF, Shaw M, Janicke H (2014). Critical theory as an approach to the ethics of information security. Sci Eng Ethics.

[116] Samsuri S, Ismail Z, Ahmad R. Adopting a knowledge management concept in securing the privacy of electronic medical record systems. Adv Intelligent Syst Comput. 2013:547–58.

[117] Hazin R, Brothers KB, Malin BA, Koenig BA, Sanderson SC, Rothstein MA (2013). Ethical, legal, and social implications of incorporating genomic information into electronic health records. Genet Med.

[118] Williams RL, Taylor JF (2016). Four steps to preserving adolescent confidentiality in an electronic health environment. Curr Opin Obstet Gynecol.

[119] Kluge EHW (1994). Health information, privacy, confidentiality and ethics. Int J Bio-Med Comput.

[120] Kluge EHW (1994). Health information, the fair information principles and ethics. Methods Inf Med.

[121] Alanazi HO, Jalab HA, Alam GM, Zaidan BB, Zaidan AA (2010). Securing electronic medical records transmissions over unsecured communications: an overview for better medical governance. J Med Plants Res.

[122] Ozair FF, Jamshed N, Sharma A, Aggarwal P (2015). Ethical issues in electronic health records: a general overview. Perspect Clin Res.

[123] Meslin EM, Alpert SA, Carroll AE, Odell JD, Tierney WM, Schwartz PH (2013). Giving patients granular control of personal health information: using an ethics ‘Points to Consider’ to inform informatics system designers. Int J Med Inform.

[124] Meslin EM, Schwartz PH (2015). How bioethics principles can aid Design of Electronic Health Records to accommodate patient granular control. J Gen Intern Med.

[125] Tehrani N (2015). How Digital Health Technology Aids Physicians. Int J Biomed.

[126] McCarthy MW, de Asua DR, Gabbay E, Fins JJ (2018). Off the charts: <i>medical documentation and selective redaction in the age of transparency</i>. Perspect Biol Med.

[127] Fairweather NB, Rogerson S (2001). A moral approach to electronic patient records. Inform Health Soc Care.

[128] Mann SP, Savulescu J, Sahakian BJ. Facilitating the ethical use of health data for the benefit of society: Electronic health records, consent and the duty of easy rescue. Philosophical Transact Royal Soc A. 2016;374(2083). 10.1098/rsta.2016.0130 PMC5124071 28336803

[129] Rigby M, Draper R, Hamilton I (1999). Finding ethical principles and practical guidelines for the controlled flow of patient data. Methods Inf Med.

[130] Kluge EHW (1999). Medical narratives and patient analogs: the ethical implications of electronic patient records. Methods Inf Med.

[131] Gummadi S, Housri N, Zimmers TA, Koniaris LG (2014). Electronic medical record: a balancing act of patient safety, privacy and health care delivery. Am J Med Sci.

[132] Cato KD, Bockting W, Larson E (2016). Did i tell you that? Ethical issues related to using computational methods to discover non-disclosed patient characteristics. J Empirical Res Hum Res Ethics.

[133] Williams H, Spencer K, Sanders C, Lund D, Whitley EA, Kaye J, et al. Dynamic Consent: A Possible Solution to Improve Patient Confidence and Trust in How Electronic Patient Records Are Used in Medical Research. JMIR Med Inform. 2015;3(1). 10.2196/medinform.3525 PMC4319083 25586934

[134] Lee LM (2017). Ethics and subsequent use of electronic health record data. J Biomed Inform.

[135] Angst CM (2009). Protect my privacy or support the common-good? Ethical questions about electronic health information exchanges. J Bus Ethics.

[136] Kopala B, Mitchell ME (2011). Use of digital health records raises ethics concerns. JONA’s Healthcare Law Ethics Regul.

[137] Klumpp TR (2013). Electronic medical records and quality of cancer care. Curr Oncol Rep.

[138] Entzeridou E, Markopoulou E, Mollaki V (2018). Public and physician’s expectations and ethical concerns about electronic health record: benefits outweigh risks except for information security. Int J Med Inform.

[139] Cheshire WP (2014). Can electronic medical records make physicians more ethical?. Ethics Med.

[140] Ow Yong LM, Tan AWL, Loo CLK, Lim ELP (2014). Risk mitigation of shared electronic records system in campus institutions: medical social work practice in Singapore. Soc Work Health Care.

[141] Wallace IM. Is patient confidentiality compromised with the electronic health record?: a position paper. Comp Inform Nurs. 2015;33(2):58–62; quiz E1. 10.1097/CIN.0000000000000126 25532832

[142] Kaplan B (2016). How should health data be used? Privacy, secondary use, and big data sales. Camb Q Healthc Ethics.

[143] Brisson GE, Neely KJ, Tyler PD, Barnard C (2015). Should medical students track former patients in the electronic health record? An emerging ethical conflict. Acad Med.

[144] Robertson MD, Kerridge IH (2009). “through a glass, darkly”: the clinical and ethical implications of Munchausen syndrome. Med J Aust.

[145] Spencer A, Low D (2011). The challenge of the information culture for the paediatrician. Arch Dis Child.

[146] Kool L, Timmer J, Royakkers L, Van Est R. Urgent upgrade: protect public values in our digitized society. The Hague: Rathenau Instituut; 2017.

[147] Shenoy A, Appel JM (2017). Safeguarding confidentiality in electronic health records. Camb Q Healthc Ethics.

[148] Rothstein MA (2010). The hippocratic bargain and health information technology. J Law Med Ethics.

[149] Rentmeester C. Heeding humanity in an age of electronic health records: Heidegger, Levinas, and Healthcare. Nurs Philos. 2018;19(3). 10.1111/nup.12214 29785721

[150] Moros DA (2017). The electronic medical record and the loss of narrative. Cambridge Quart Healthcare Ethics.

[151] Franz B, Murphy JW (2015). Electronic medical records and the technological imperative: the retrieval of dialogue in community-based primary care. Perspect Biol Med.

[152] Berg M, Langenberg C, Vd Berg I, Kwakkernaat J (1998). Considerations for sociotechnical design: experiences with an electronic patient record in a clinical context. Int J Med Inform.

[153] Stein HF (2012). Interfaces between electronic medical record (EMR/EHR) technology and people in American medicine: insight. Imagination, and relationships in clinical practice. J Oklahoma State Med Assoc.

[154] Van der Ploeg I (2003). Positioning the patient: normative analysis of electronic patient records. Methods Inf Med.

[155] Cederberg RA, Valenza JA (2012). Ethics and the electronic health record in dental school clinics. J Dent Educ.

[156] Kluge EHW (2004). Informed consent and the security of the electronic health record (EHR): some policy considerations. Int J Med Inform.

[157] Whitehouse D, Duquenoy P. eHealth and ethics: theory, teaching, and practice. Information and Communication Technologies, Society and Human Beings: Theory and Framework. 2010. p. 454-65.

[158] Kluge EHW (1996). Professional ethics as basis for legal control of health care information. Int J Biomed Comput.

[159] Machado J, Miranda M, Abelha A, Neves J, Neves J. Modeling Medical Ethics through Intelligent Agents. IFIP Adv Inform Commun Technol. 2009:112–22.

[160] Nissenbaum H (2004). Privacy as contexctual integrity. Wash Law Rev.

[161] Davenport TH, Hongsermeier TM, Mc Cord KA (2018). Using AI to improve electronic health records. Harv Bus Rev.

[162] Zhou L, Blackley SV, Kowalski L, Doan R, Acker WW, Landman AB (2018). Analysis of errors in dictated clinical documents assisted by speech recognition software and professional transcriptionists. JAMA Netw Open.

[163] Zheng Y, Ding X, Poon CCY, Lo BPL, Zhang H, Zhou X (2014). Unobtrusive sensing and wearable devices for health informatics. IEEE Trans Biomed Eng.

[164] Beauchamp TL, Childress JF (2012). Principles of biomedical ethics. 7th edition ed.

[165] Mittelstadt BD, Allo P, Taddeo M, Wachter S, Floridi L (2016). The ethics of algorithms: mapping the debate. Big Data Soc.

[166] Royakkers L, Timmer J, Kool L, van Est R (2018). Societal and ethical issues of digitization. Ethics Inf Technol.

