# Supplementary material for: Examination and diagnosis of electronic patient records and their associated ethics: a scoping literature review
Source: BMC Med Ethics. 2020 Aug 24;21:76. doi: 10.1186/s12910-020-00514-1 (PMC7446190; doi:10.1186/s12910-020-00514-1)
Supplement: Supplementary file 1 — Additional file 1. Overview of sources. [file 12910_2020_514_MOESM1_ESM.docx]

# **Appendix A: Overview of sources**

The below table provides an overview of the articles discussed in the literature review. Below is a short explanation of how to read this overview.

**Functionality**

An electronic patient record (EPR) can have many functions but sometimes an article discusses only one of these functionalities. An article, for example, may discuss the opportunities and challenges associated with sharing medical information with courts for legal purposes. The author of that article may acknowledge that the primary use of an EPR is the improvement of care but chose to discuss the use of EPRs by courts. This column indicates the particular functionality or purpose discussed in the article regardless of other possible functionalities.

**Type of user**

Similarly to the above, an EPR can be used by a variety of users. This column identifies the users as discussed in the article. Take for example an article which discusses ethical issues around the use of clinical records for research purposes. The main users of the EPR for this purpose are researchers, which does not exclude the possibility that the main users of the same record in a clinical setting would be healthcare professionals. The type of users only indicates the users discussed in the article.

**Ethical issue:**

The main normative point in the text is condensed to one sentence or a few values to provide a short overview of what this text aims to convey.

**Rights and Duties:**

The header ‘rights’ identifies the stakeholders the article has identified whose rights need to be protected. An article may mention patients as the stakeholder group whose rights need to be protected. Similarly, the column with the header ‘duties’ contains the stakeholder group who is responsible to protect the rights. So an article may argue that the patients (in the column ‘rights’) needs protection and the clinicians (in the column ‘duties’) need to protect these rights. Cells in the table are sometimes left blank: we have left these blank because the source did not specify the actor. For example, an article may argue that patients have rights to privacy without specifying the stakeholder responsible for ensuring these rights.

| **Author** | **Functionality** | **Type of user** | **Ethical issue** | **Rights** | **Duties** |
| --- | --- | --- | --- | --- | --- |
| (1) | Genomic information into the electronic medical record | Clinician | Main value is privacy - who should be informed about genetic findings? How to communicate these findings to patients? | Patient | Multi-faceted and interdisciplinary approach |
| (2) | clinical data exchange - sharing with patient | Clinician | Tensions between the qualitative, unstructured nature of medical language and the logical, structured nature of EHR technology | Patient | Unspecified |
| (3) | clinical data exchange - sharing with court | Clinician | Confidentiality and patient control over content for legal purposes and the clinicians duty to accurately populated the record | Patient | Clinician |
| (4) | clinical data exchange - clinical decision making systems (genomic data) | Clinician | Confidentiality issues around improper use of EPR. Unjust economic or population-based barriers to treatment arising from genomic data from EPRs. | Patient | Ethics boards or other governance bodies |
| (5) | Clinical record | Developers/IT structure | Privacy and security in technology design | Patient | Developer |
| (6) | Training/education | Clinicians/Patients | Deskilling of students in capturing information. Data management skills for students needed. | Patient/ students | Interdisciplinary |
| (7) | Clinical record and other functionality | Clinicians/other such as insurers | Balancing of public good and individual interests and rights in privacy and confidentiality | Patient/ Society | Patients/ Society |
| (8) | Clinical record/ research | Developers | Privacy and confidentiality regarding research | Patient | Developers |
| (9) | Clinical record | Clinicians (Psychologists) | Psychologists practicing in a hospital or academic medical settings have complex relationships and work in multidisciplinary teams while they need to maintain confidentiality and consent. | Patients/team member | Psychologists |
| (10) | Clinical record | Researchers | Biases in EHR data research and the consequences for health equity | Patients | Researchers |
| (11) | Clinical record | Clinicians | Rights and duties around safe and secure EHR technology | Patients | Unknown |
| (12) | Clinical record | Clinicians (Primary care) | EMR changes workflows, privacy issues becuase access to EMR by support staff and IT support, and a lack of appropriate governance | Clinicians | Uncleare |
| (13) | Clinical record | Clinicians (geriatrician) | Geriatricians should do good and avoid harm and therefore thoroughly vet EMR technology | Patients | geriatrician |
| (14) | Clinical record | Clinicians/Patients | The challenges around sharing EPRs with patients: concerns about cost and security concerns, the assigning of responsibilities and rights, and tensions between access to data and access to physicians | Patient | Platform developers |
| (15) | Clinical record | Clinicians | Addressing privacy obstacles to create usable but privacy safe EPR technology | Patients/ clinicians | Policymakers |
| (16) | Clinical record | Clinicians | Functionality which enables carelessness and harmful shortcuts rather than reap the timesaving benefits | patients | Developers/Clinicians |
| (17) | Clinical record | Clinicians | Tension between the rights of the patient to control the access to their EHRs’ data and the needs of healthcare professionals for complete and accurate health data to make the best treatment decisions for their patients | Patients/healthcare professionals | healthcare professionals |
| (18) | Educational | Academic hospitals | Ethical framework for tracking for educational purposes: considers privacy and consent vs the benefit for students. | Students: Benefits | Academic hospitals |
| (19) | Educational | Academic hospitals | Conflicts between medical education and patient privacy. | Patient: Autonomy, privacy, security Students: benefits. Society: public good | Academic hospitals |
| (20) | Research | Researchers | A biobank-specific Ethics Committee to guarantee access controls and security measures and avoid breach of privacy and of stigmatization | Patients/healthcare providers | Policy |
| (21) | Clinical record | Clinicians | Trust from patients and doctors requires adherence to legal, ethical and social values | Patients | Technical, legal, ethical barriers in implementing technology |
| (22) | Data analytics | Researchers | Balancing the rights of patients (following the Belmont principles: autonomy, justice, beneficence) with the public good | Patient | researchers and ethical review boards |
| (23) | Educational | Dentists | The use of EHRs by dental schools and concerns around the doctor-patient relationship, privacy and professional conduct and the management of ethical issues. | Patients | Dental schools |
| (24) | Clinical record | Clinicians | A human will always need to be in the loop even with automated processes as automation can never adequately substitute virtue | Patients | Clinicians |
| (25) | Clinical record | Clinicians | Threats to privacy and confidentiality, patients should opt-in and give informed consent regarding information about metal health. | patients | psychotherapist |
| (26) | Clinical Record | Clinicians | EMRs will transform how clinicians think, write, and speak about patients. The narrative style of recording information will be lost. | Clinicians | Clinicians |
| (27) | Patient portal functionality | Patients/Clinicians | Benefits of patient access to data against the risks of possible misinterpretation and stress of hearing results without adequate clinical guidance. Issues around timing of information. | Patients | Clinicians |
| (28) | Clinical Record | Clinicians | the loss of skills and knowledge by reducing patients to sets of data points, shift of power to administrators, deprivation of professional autonomy, increased workload for clinicians, too much attention for institutional priorities | Clinicians/Patients | Unclear |
| (29) | Clinical Record | Clinicians | Applications of EHR and social media for  diabetes surveillance and issues around privacy, biases and completeness | Clinicians/Patients | Development of EHRs |
| (30) | Clinical Record | Clinicians | Public and clinicians believe that EHRs will have a positive impact on health and quality of care. Although they could be a burden on clinicians’ time, resources, and workflow. Clinicians are more restrictive concerning access than patients. Clinicians are in favour of more centralized EHR systems. | Patients | Unspecified |
| (31) | Clinical record | Clinicians | Selection of challenges associated with EHRs in developing countries: privacy, security breaches, system Implementation and poor fit with workflow or poor user interface design, data inaccuracies | Patients | physicians, technology professionals, ethicists, administrative personnel, and patients |
| (32) | Unspecified | Unspecified | A moral approach to EPRs based on: beneficence, non-maleficence, autonomy, justice | Patients | Unspecified |
| (33) | Clinical record | Clinicians | EHR can affect the physician-patient relationship: secondary uses may affect trust negatively but EHR can promote trust through transparency and communication enhancements | Patients | Physicians |
| (34) | Clinical Record | Clinicians | Stresses the value of participation, solidarity, and community ownership in the development of EHR technology. | Patients | Development of EHRs/clinicians |
| (35) | Learning health system | Clinicians | EPRs move the national health economy towards a learning system | Public good | Patient population/healthcare sector |
| (36) | Patient participation | Clinicians/Patients | National EPR systems need: clarification of key ethical concerns, interests stakeholders in those concerns, and consensus on addressing key ethical concerns. Key concerns are privacy and a conflict of interests | Patients | Government/developers |
| (37) | manage patient care | Clinicians/Patients | A set of privacy specific heuristics aimed at patient control over access to data | Patients | Unspecified |
| (38) | streamline care | Clinicians/Patients | Policymakers to become familiar with the intentions of the multiple stakeholders before starting with digitalisation | Patients | Policy-makers |
| (39) | Improve public healthcare | Clinicians/Patients | EPRs improve public health and clinicians and patients therefore have a moral obligation to support EPRs | public healthcare | Patient/Clinicians |
| (40) | Improvement healthcare and healthcare management | Clinicians/Patients | Balance between challenges and opportunities of EPR: Challenges such as privacy, system and data entry error and opportunities such as improvement in the quality of care, and support of research and compliance | public health, physicians, patients | policy makers |
| (41) | Clinical record | Clinicians | Identify burdensome IT changes to the clinician which lower quality of patient care; and methods for clinicians to become more IT aware | Clinician/ patient | Educate clinicians |
| (42) | Clinical record | Clinicians | Implementation of genomics requires a balance between improving healthcare and reducing potential harms to patient autonomy, equity and confidentiality | Patients | Physician/ healthcare systems |
| (43) | Clinical record | Clinicians | Challenges around data capture such as data entry errors, bias, focus on administration rather than clinical uses | public health | public health researchers and surveillance authorities, public health community |
| (44) | Research | Researchers | Suggestion to use data of deceased patients and description of ethical ways to create this type of research platform | public health |  |
| (45) | clinical record | Healthcare providers | Building trust in the design of a national EHR system analysis roles while focusing on privacy concerns |  |  |
| (46) | clinical record | Healthcare providers | Defines requirements for confidentiality, integrity, and patient control over data. | patients | systems |
| (47) | medical research | Clinicians/Patients | A broad range of ethical, legal and technical barriers towards mining EHR data. | patients | all stakeholders |
| (48) | data mining, and secondary use | unspecified | Selling data and the opportunities for healthcare and costs and challenges such as confidentiality, transparency and consent. | patients | Policy |
| (49) | Clinical record | Clinicians | Challenges from security breaches: uphold trust in the patient-physician relationship, to prevent harms to patients, and to respect patients’ autonomy | patients | physicians, medical practices, and health care institutions |
| (50) | Clinical record | health information professionals | Electronic patients records require a paradigm change from property model to something more suitable for the digital age based on fair information principles | Patients/ clinicians | Policymakers |
| (51) | Clinical record | health information professionals | Electronic patients records require a paradigm change from property model to something more suitable for the digital age based on fair information principles, privacy and confidentiality | Patients/ clinicians | Policymakers |
| (52) | clinical record | health information professionals/health organisations | Code of professional ethics for Health Information Professionals | patients | health information professionals |
| (53) | clinical record | Health Information Professionals | Patient have a right to informed consent, and the professionals and institutions have a duty to ensure this right. However, right to informed consent is limited. An appropriately structured security architecture will recognise these limits | patients | Trusted authority |
| (54) | unknown | unknown | The limitations of requiring informed consent for secondary usage: values at play are autonomy and beneficence | patients, researcher, public good | Policy maker |
| (55) | Clinical record | Health information professionals | Development of uniform, ethical and internationally enforceable standards for EPRs and healthcare information professionals. | Patients | Health Information Professionals |
| (56) | Clinical Record | Clinicians | A call to arms for the identification of relevant ethical principles, and the development of regulations | Patients | Ethicists |
| (57) | Clinical record | Clinical | Clinicians have an ethical obligation to consider cost and effectiveness when introducing new medications, diagnostic procedures, and other techniques | Unspecified | Clinicians |
| (58) | EMR/EHR/PP | Patient, healthcare professional, organisation | Weighing of privacy and security breaches, costs, data inaccuracies and implementation barriers against benefits to public health | Patients/public good | Healthcare organisations, Patients right to public health |
| (59) | Clinical record | Clinician/researcher | Transformation of clinical photography brings new ethical, legal, and social concerns, such as privacy, informed consent, standardisation for quality photos, security, and humiliation and shame around explicit photography | Patients | Healthcare staff |
| (60) | Clinical record | Clinician/researcher | Conflicts among the ethical principles of beneficence, autonomy, fidelity, and justice resulting from EHRs | unspecified | unspecified |
| (61) | Patient portal to radiology | Patients/Clinicians | Benefits of patients reviewing their own radiology reports: improved understanding of their own health, patient-physician communication, and shared decision-making promote,. Challenges may be patient anxiety and longer patient-physician interactions. | unspecified | radiologists/investments |
| (62) | Clinical | Healthcare professionals | Benefits and challenges of using EHR for research and health services: quality improvement and learning health system. Other challenges include data validity, harms from undesirable use, patient, biases, and the necessity of ethics training for personnel. | Patients/public good | Clinicians/researchers, policy makers |
| (63) | Clinical | Healthcare professionals | Rawlsian model: risk selection by health insurers is morally impermissible |  | Policy maker |
| (64) | Unknown | Unknown | A first step towards framework for an integrated approach to ethics and privacy | Patients | Systems |
| (65) | Clinical record | Radiologists | Physician’s professionals is needed for the benefits to outweigh the harms. Harms are to time and resource drains, transparency, confidentiality, direct advertisements of new drugs, and the quality of outsourced radiology readings. | Patients | physicians’ professionalism |
| (66) | Secondary use | Researcher | Types of consent, opting out or anonymisation, and duties to benefit others with the use of data for health research. | Patients/public good | Researchers |
| (67) | Clinical record | Clinical | The EPR should provide benefits to primary user, i.e. clinicians should not be burdened by EPR developed for secondary research. The technology should be conceptualized as a tool for clinicians and adjusted to the needs of clinicians instead of being overly structured. | Clinicians | Design system |
| (68) | Clinical record | Clinical | Behavior of machines toward human users, and perhaps other machines as well should be ethically acceptable. | Human Users | Design system |
| (69) | Research | Researchers | The ethical tensions between advancing biomedical research and protecting patient privacy | Patient/Society | Patients/ healthcare professionals |
| (70) | Clinical record | Nurses | Ethical challenges using EPRs in nursing practice | Nurses | Multidisciplinary approach |
| (71) | Clinical record | Clinical | What to capture on medical charts and what to show patients and challenges arising from these questions: timing of information, clinician’s freedom to ask difficult questions without scrutiny, the importance of timing when relaying information for the clinician patient relationship, and education for students and clinicians about these issues | Patients | procedural and technological mechanisms |
| (72) | Research | Researchers | Challenges when sharing genomic data with researchers and institutions. Processes to protect privacy and security and prevent discrimination. | HCPs and policy makers | Unspecified |
| (73) | Clinical record | Clinical record | Policies to protect confidentiality, privacy, and security of genetic/genomic test information | Patients | policy developers and health care professionals |
| (74) | Educational | Students | Privacy and confidentiality issues associated with the use of clinical EPR data by students for the purposes of learning | Students | Educators |
| (75) | Clinical (linking data) | Clinical | Shared EHR can lead to unethical disclosure of personal health information: issues around confidentiality, consent and the involvement of the private sector are examined | patients | policy |
| (76) | Clinical record | Clinical | Confidentiality should be presumed, when a patient is incapable to provide consent because of the sensitivity of EPR data. | patients | policy |
| (77) | Health platform | providers and insurers/patients/researchers | Granular control to apply ethical principles including respect for autonomy, beneficence, non-maleficence and justice | patients | tool design |
| (78) | clinical encounter | clinicians | Ethics should guide the design of electronic health records: respect for autonomy, beneficence, non-maleficence, and justice | patients | clinicians |
| (79) | clinical encounter | Nurses | The role of nurses for protecting privacy and confidentiality and ensure benefits when using EPRs | patients | Nurses |
| (80) | clinical encounter | clinicians | Ways for care providers to protect patient confidential and only share information with the authorisation of the patient | patients | Institutions |
| (81) | clinical encounter | clinicians | Confidentiality and security | Patients | Health providers |
| (82) | clinical encounter | clinicians | Pediatric population with mental health issues and risks related to EHR access, confidentiality, privacy and professional collaboration | patients | professionals and organizations |
| (83) | Educational | health information professionals | EPR software to educate students becoming health information professionals | patients | health information professionals |
| (84) | clinical encounter | health information professionals | Familiarity with guiding ethical principles was important to create patient centric EPRs in Singapore | patients | health information professionals |
| (85) | clinical encounter | clinicians | Beneficence dictates that benefits of adoption should significantly outweigh burdens and current standards do not sufficiently guarantee that | patients/ clinicians | System, policy makers, clinical users |
| (86) | clinical encounter | clinicians | Concerns about the benefits because of errors with data entry, privacy, and patient - clinician interaction. | patients | Clinicians and healthcare organisations |
| (87) | clinical encounter | clinicians | Cultural challenges required before integration EPRs | Patients | Unspecified |
| (88) | Clinical record | Clinicians | A human centred or value centred design is required to ensure patients are presented appropriate, and have agency and identity. | Patients | Unspecified (those responsible for the design of EPRs) |
| (89) | clinical encounter/ write your own record | Clinicians/patients | Patient’s access to information without context, the patients’ responsibility for modifying health record | health professionals and patients | health professionals and patients |
| (90) | predictive analytic (e-HPA) applications | Unknown | Ethics as part of framework to guide predictive algorithms based on EPRs | patients | developers of EHR |
| (91) | clinical encounter | nurses | Templates in EPR systems may erode respect for humanity and affect the nurse–patient relationships | patients | nurses |
| (92) | clinical encounter | Clinicians | Ethical access control mechanisms for electronic patient records are required and should be published | Patients | Unspecified (those responsible for publishing algorithms) |
| (93) | clinical encounter | clinicians | EMRs unlikely to reveal the kind of sensitive mental health information required for some patients with mental health issues. Cost savings insufficient justification for injustices and privacy breaches mental health patients will suffer. | patients | clinicians |
| (94) | clinical encounter | clinicians | The Hippocratic oath established privacy and confidentiality but needs to be reformulated regarding because of EHRs, as privacy concerns and the clinician-patient relationship has changed. More responsibilities for patient to maintain control of health | patients/public health | clinicians |
| (95) | clinical encounter | clinicians | Compelled authorizations: in current form insufficient to protect patient privacy | patients/public health | clinicians |
| (96) | clinical encounter | clinicians | Guidelines for breaches of electronic medical records | patients/public health | clinicians |
| (97) | unknown | unknown | Ethics and culture are part of human centered design: privacy a component when designing EMRs | Patients | Design of EHR |
| (98) | clinical encounter | clinicians | Chart notes within EHRs system (EPIC) to improve understanding of ethical concepts | Patients/Staff | CE consultation services |
| (99) | clinical encounter | clinicians | Clinicians should prevent breaches of the EHR and, respond appropriately to breaches if necessary | patients | clinicians |
| (100) | clinical encounter | clinicians | EHR issues need to be addressed globally to protect ethical concerns such as confidentiality and consent | Patients | EHR System/Policy makers |
| (101) | clinical encounter | clinicians | Institutional model setting access limitations to protect patient confidentiality | patient | institutional |
| (102) | clinical encounter | clinicians | Incorporating genetic data into EHRs: ethical issues can be summarised as duties to maximize good and minimise harm and respect autonomy. | patient | developers |
| (103) | clinical encounter | clinicians | Pediatric records: the sale of records and ownership and re-identification issues, access of parents to records of adolescents, and matching records across institutes | Patient | Policy (setting up a national forum) |
| (104) | clinical encounter | clinicians | Protection of a minor's right to privacy in the South African healthcare context | Minor | Law makers |
| (105) | clinical encounter | clinicians | Students/pediatric psychologists should become familiar with confidentiality beyond the American Psychological Association’s ethical guidelines, understand their legal duties and determine what takes precedent. | Patients | students/pediatric psychologists |
| (106) | clinical encounter | Researchers | Ethical concerns around Learning Health System and a need for transparency: even though research is conducted with de-identified data some patients find consent required and breaches pose a risk |  |  |
| (107) | clinical encounter | Pediatricians | Pediatricians should take responsibility for the accuracy and timeliness of data and use technology to change care | minors | Pediatricians |
| (108) | clinical encounter | Clinicians | Nationwide personal EHR: ethical questions that need to be addressed | patients | Policy |
| (109) | clinical encounter | Clinicians | Critical theory to understand and address possible ethical issues such as power, ownership, legitimacy and justification of action, emancipation, the good life and other implications for the individual | patients | NHS |
| (110) | clinical encounter | Clinicians | Human relationships should be improved instead of impoverished when using EMRs. EMRs should not lead to conveyer belt | Patients | Clinicians |
| (111) | clinical encounter | Clinicians | Concerns around designing and implementing EHR technology in hospitals: Nurture the everyday knowledge of nursing staff and the patient | nurses/patients | System design |
| (112) | Primarily billing, administrative, and regulatory elements. Patient and physicians stories seondary | Clinicians | The patient should be prioritised. The impact of EHRs on the patient–physician relationships, time pressure, human skills, critical thinking, accurate complete information, patient privacy/confidentiality, access to information, the digital divide | patient–physician | system |
| (113) | improving healthcare | Clinicians/patients | EHR technology allows for enhanced doctor/patient experience. Medical errors and breaches of patient privacy should be avoided | healthcare |  |
| (114) | clinical encounter | Clinicians | Neuropsychologists will face ethical issues around confidentiality and privacy: they will need to anticipate ethical issues, avoid, address and aspire | Patients | Administration |
| (115) | clinical encounter | Clinicians | Ownership of the computerized medical record, access to medical information, medical record brokering, confidentiality, and research and development | Patients | System |
| (116) | clinical encounter | Clinicians | Nurses need to protection patient privacy they need to evaluate, educate, and monitor end users | patient | nurses |
| (117) | clinical encounter | Clinicians | Scribes may be a solution for physicians, but patients’ confidentiality should not be protected. | patients | physicians |
| (118) | clinical encounter | Clinicians | Benefits and risks around capturing content in EHR through technology: improvement and enhanced efficiency care and communication but copying can also cause data errors and diminish trustworthiness | patient | clinicians |
| (119) | clinical encounter | Clinicians | A lack of understanding of EHR data places certain patients at a disadvantage: There may be an impact on equality and equity generally | patient | clinicians |
| (120) | clinical encounter | School nurses | Confidentiality, privacy and control of information in a tradeoff between free and paid EHR offerings | school nurse | clinicians |
| (121) | clinical encounter | Clinicians | Understanding best practices for adolescent health care services prescribe allows an adoption of EHR that respects confidentiality | adolescents | clinicians |
| (122) | Health research | Clinicians | Dynamic consent model to maintain public trust to leverage data for research |  |  |
| (123) | Patient participation; promoting communication and data use | Patients | Balancing patient interest with vendor interests. Benefits may not be distributed equitable. EHR may take up much valuable time of patient and doctor. Balancing the doctors want for accurate information with the patient’s want to control the information. Patient’s ability to assume responsibility for an EHR. |  |  |

# **Bibliography**

1. Nishimura AA, Tarczy-Hornoch, P., Shirts, B. H. Pragmatic and Ethical Challenges of Incorporating the Genome into the Electronic Medical Record. Current genetic medicine reports. 2014;2(4):201-11.

2. Roberts A. Language, Structure, and Reuse in the Electronic Health Record. AMA journal of ethics. 2017;19(3):281-8.

3. Accordino R, Kopple-Perry N, Gligorov N, Krieger S. The medical record as legal document: When can the patient dictate the content? An ethics case from the Department of Neurology. Clinical Ethics. 2014;9(1):53-6.

4. Al Mallah A, Guelpa, P., Marsh, S., van Rooij, T. Integrating genomic-based clinical decision support into electronic health records. Personalized medicine. 2010;7(2):163-70.

5. Alanazi HO, Jalab HA, Alam GM, Zaidan BB, Zaidan AA. Securing electronic medical records transmissions over unsecured communications: An overview for better medical governance. Journal of Medicinal Plants Research. 2010;4(19):2059-74.

6. Altman M. The Clinical Data Repository: A Challenge to Medical Student Education. Journal of the American Medical Informatics Association. 2007;14(6):697-9.

7. Angst CM. Protect my privacy or support the common-good? ethical questions about electronic health information exchanges. Journal of Business Ethics. 2009;90(SUPPL. 2):169-78.

8. Anuradha C, Babu PBR. Securing privacy for confidential databases using anonymization. Middle - East Journal of Scientific Research. 2012;12(12):1792-5.

9. Ashton K, Sullivan, A. Ethics and Confidentiality for Psychologists in Academic Health Centers. Journal of clinical psychology in medical settings. 2018;25(3):240-9.

10. Hollister B, Bonham, V. L. Should Electronic Health Record-Derived Social and Behavioral Data Be Used in Precision Medicine Research? AMA journal of ethics. 2018;20(9):E873-80.

11. Bakker A. Digest of the discussion group sessions. Realising Security of the Electronic Record. International journal of medical informatics. 2004;73(3):325-31.

12. Balka E, Tolar, M. Everyday ethical dilemmas arising with electronic record use in primary care. Studies in health technology and informatics. 2011;169:285-9.

13. Barber A. Computers for physicians: Never do harm. Care Management Journals. 2012;13(4):194-9.

14. Beard L, Schein R, Morra D, Wilson K, Keelan J. The challenges in making electronic health records accessible to patients. Journal of the American Medical Informatics Association. 2012;19(1):116-20.

15. Ben-Assuli O. Electronic health records, adoption, quality of care, legal and privacy issues and their implementation in emergency departments. Health policy (Amsterdam, Netherlands). 2015;119(3):287-97.

16. Bernat JL. Ethical and quality pitfalls in electronic health records. Neurology. 2013;80(11):1057-61.

17. Bhuyan SS, Bailey-DeLeeuw S, Wyant DK, Chang CF. Too Much or Too Little? How Much Control Should Patients Have Over EHR Data? Journal of medical systems. 2016;40(7).

18. Brisson GE, Barnard, C., Tyler, P. D., Liebovitz, D. M., Neely, K. J. A Framework for Tracking Former Patients in the Electronic Health Record Using an Educational Registry. Journal of general internal medicine. 2018;33(4):563-6.

19. Brisson GE, Neely, K. J., Tyler, P. D., Barnard, C. Should medical students track former patients in the electronic health record? An emerging ethical conflict. Academic medicine : journal of the Association of American Medical Colleges. 2015;90(8):1020-4.

20. Caenazzo L, Tozzo, P., Borovecki, A. Ethical governance in biobanks linked to electronic health records. European review for medical and pharmacological sciences. 2015;19(21):4182-6.

21. Casanovas P, Mendelson D, Poblet M. A Linked Democracy Approach for Regulating Public Health Data. Health and Technology. 2017;7(4):519-37.

22. Cato KD, Bockting, W., Larson, E. Did I Tell You That? Ethical Issues Related to Using Computational Methods to Discover Non-Disclosed Patient Characteristics. Journal of empirical research on human research ethics : JERHRE. 2016;11(3):214-9.

23. Cederberg RA, Valenza, J. A. Ethics and the electronic health record in dental school clinics. Journal of dental education. 2012;76(5):584-9.

24. Cheshire WP. Can electronic medical records make physicians more ethical? Ethics and Medicine. 2014;30(3):135-41.

25. Clemens NA. Privacy, consent, and the electronic mental health record: The person vs. the System. Journal of Psychiatric Practice. 2012;18(1):46-50.

26. Moros DA. The Electronic Medical Record and the Loss of Narrative. Cambridge quarterly of healthcare ethics : CQ : the international journal of healthcare ethics committees. 2017;26(2):328-31.

27. Davis KA, Smith LB. Ethical Considerations about EHR-Mediated Results Disclosure and Pathology Information Presented via Patient Portals. AMA journal of ethics. 2016;18(8):826-32.

28. de Ruiter HP, Liaschenko, J., Angus, J. Problems with the electronic health record. Nursing philosophy : an international journal for healthcare professionals. 2016;17(1):49-58.

29. Eggleston EM, Weitzman ER. Innovative uses of electronic health records and social media for public health surveillance. Current Diabetes Reports. 2014;14(3).

30. Entzeridou E, Markopoulou, E., Mollaki, V. Public and physician's expectations and ethical concerns about electronic health record: Benefits outweigh risks except for information security. International journal of medical informatics. 2018;110:98-107.

31. Ozair FF, Jamshed, N., Sharma, A., Aggarwal, P. Ethical issues in electronic health records: A general overview. Perspectives in clinical research. 2015;6(2):73-6.

32. Fairweather NB, Rogerson S. A moral approach to electronic patient records. Informatics for Health and Social Care. 2001;26(3):219-34.

33. Francis LP. The physician-patient relationship and a national health information network. Journal of Law, Medicine and Ethics. 2010;38(1):36-49.

34. Franz B, Murphy JW. Electronic medical records and the technological imperative: The retrieval of dialogue in community-based primary care. Perspectives in biology and medicine. 2015;58(4):480-92.

35. Friedman C, Rigby M. Conceptualising and creating a global learning health system. International journal of medical informatics. 2013;82(4):e63-e71.

36. Fry CL, Spriggs M, Arnold M, Pearce C. Unresolved Ethical Challenges for the Australian Personally Controlled Electronic Health Record (PCEHR) System: Key Informant Interview Findings. AJOB Empirical Bioethics. 2014;5(4):30-6.

37. Furano RF, Kushniruk, A., Barnett, J. Deriving a Set of Privacy Specific Heuristics for the Assessment of PHRs (Personal Health Records). Studies in health technology and informatics. 2017;234:125-30.

38. Garrety K, McLoughlin, I., Wilson, R., Zelle, G., Martin, M. National electronic health records and the digital disruption of moral orders. Social science & medicine (1982). 2014;101:70-7.

39. Goodman KW. Ethics, information technology, and public health: New challenges for the clinician-patient relationship. Journal of Law, Medicine and Ethics. 2010;38(1):58-63.

40. Gummadi S, Housri, N., Zimmers, T. A., Koniaris, L. G. Electronic medical record: a balancing act of patient safety, privacy and health care delivery. The American journal of the medical sciences. 2014;348(3):238-43.

41. Haig SV. Ethical choice in the medical applications of information theory. Clinical Orthopaedics and Related Research. 2010;468(10):2672-7.

42. Hazin R, Brothers, K. B., Malin, B. A., Koenig, B. A., Sanderson, S. C., Rothstein, M. A., Williams, M. S., Clayton, E. W., Kullo, I. J. Ethical, legal, and social implications of incorporating genomic information into electronic health records. Genetics in medicine : official journal of the American College of Medical Genetics. 2013;15(10):810-6.

43. Hoffman S, Podgurski, A. Big bad data: law, public health, and biomedical databases. The Journal of law, medicine & ethics : a journal of the American Society of Law, Medicine & Ethics. 2013;41 Suppl 1:56-60.

44. Huser V, Cimino JJ. Don't take your EHR to heaven, donate it to science: Legal and research policies for EHR post mortem. Journal of the American Medical Informatics Association. 2014;21(1):8-12.

45. Iacovino L, Reed B. Recordkeeping research tools in a multi-disciplinary context for cross-jurisdictional health records systems. Archival Science. 2008;8(1):37-68.

46. Wainer J, Campos, C. J., Salinas, M. D., Sigulem, D. Security requirements for a lifelong electronic health record system: an opinion. The open medical informatics journal. 2008;2:160-5.

47. Jensen PB, Jensen LJ, Brunak S. Mining electronic health records: Towards better research applications and clinical care. Nature Reviews Genetics. 2012;13(6):395-405.

48. Kaplan B. How Should Health Data Be Used? Privacy, Secondary Use, and Big Data Sales. Cambridge Quarterly of Healthcare Ethics. 2016;25(2):312-29.

49. Kim D, Schleiter K, Crigger BJ, McMahon JW, Benjamin RM, Douglas SP, et al. A physician's role following a breach of electronic health information. The Journal of clinical ethics. 2010;21(1):30-5.

50. Kluge EHW. Health information, the fair information principles and ethics. Methods of information in medicine. 1994;33(4):336-45.

51. Kluge EHW. Health information, privacy, confidentiality and ethics. International Journal of Bio-Medical Computing. 1994;35(SUPPL.):23-7.

52. Kluge EHW. Fostering a security culture: A model code of ethics for health information professionals. International journal of medical informatics. 1998;49(1):105-10.

53. Kluge EH. Informed consent and the security of the electronic health record (EHR): some policy considerations. International journal of medical informatics. 2004;73(3):229-34.

54. Kluge EHW. Informed consent to the secondary use of EHRs: Informatic rights and their limitations. Studies in health technology and informatics2004. p. 635-8.

55. Kluge EHW. Professional ethics as basis for legal control of health care information. International Journal of Bio-Medical Computing. 1996;43(1-2):33-7.

56. Kluge EH. Medical narratives and patient analogs: the ethical implications of electronic patient records. Methods of information in medicine. 1999;38(4-5):253-9.

57. Klumpp TR. Electronic medical records and quality of cancer care. Current Oncology Reports. 2013;15(6):588-94.

58. Kopala B, Mitchell ME. Use of digital health records raises ethics concerns. JONA's Healthcare Law, Ethics, and Regulation. 2011;13(3):84-9.

59. Lakdawala N, Fontanella, D., Grant-Kels, J. M. Ethical considerations in dermatologic photography. Clinics in dermatology. 2012;30(5):486-91.

60. Layman EJ. Ethical issues and the electronic health record. The health care manager. 2008;27(2):165-76.

61. Lee CI, Langlotz CP, Elmore JG. Implications of Direct Patient Online Access to Radiology Reports Through Patient Web Portals. Journal of the American College of Radiology. 2016;13(12):1608-14.

62. Lee LM. Ethics and subsequent use of electronic health record data. Journal of biomedical informatics. 2017;71:143-6.

63. Lercher A. A social contract for health information. Journal of Information Ethics. 2008;17(2):35-45.

64. Liyanage H, Liaw ST, Di Iorio CT, Kuziemsky C, Schreiber R, Terry AL, et al. Building a Privacy, Ethics, and Data Access Framework for Real World Computerised Medical Record System Data: A Delphi Study. Contribution of the Primary Health Care Informatics Working Group. Yearbook of medical informatics. 2016(1):138-45.

65. Lo B. Professionalism in the age of computerised medical records. Singapore Medical Journal. 2006;47(12):1018-22.

66. Lowrance WW. Learning from Experience: Privacy and the Secondary Use of Data in Health Research. Journal of Biolaw and Business. 2003;6(4):30-60.

67. Berg M, Langenberg, C., vd Berg, I., Kwakkernaat, J. Considerations for sociotechnical design: experiences with an electronic patient record in a clinical context. International journal of medical informatics. 1998;52(1-3):243-51.

68. Machado J, Miranda M, Abelha A, Neves J, Neves J. Modeling Medical Ethics through Intelligent Agents. IFIP Advances in Information and Communication Technology2009. p. 112-22.

69. Mann SP, Savulescu J, Sahakian BJ. Facilitating the ethical use of health data for the benefit of society: Electronic health records, consent and the duty of easy rescue. Philosophical Transactions of the Royal Society A: Mathematical, Physical and Engineering Sciences. 2016;374(2083).

70. McBride S, Tietze M, Robichaux C, Stokes L, Weber E. Identifying and addressing ethical issues with use of electronic health records. Online Journal of Issues in Nursing. 2018;23(1).

71. McCarthy MW, de Asua, D. R., Gabbay, E., Fins, J. J. Off the Charts: <i>Medical documentation and selective redaction in the age of transparency</i>. Perspectives in biology and medicine. 2018;61(1):118-29.

72. McGuire AL, Basford, M., Dressler, L. G., Fullerton, S. M., Koenig, B. A., Li, R., McCarty, C. A., Ramos, E., Smith, M. E., Somkin, C. P., Waudby, C., Wolf, W. A., Clayton, E. W. Ethical and practical challenges of sharing data from genome-wide association studies: the eMERGE Consortium experience. Genome research. 2011;21(7):1001-7.

73. McGuire AL, Fisher, R., Cusenza, P., Hudson, K., Rothstein, M. A., McGraw, D., Matteson, S., Glaser, J., Henley, D. E. Confidentiality, privacy, and security of genetic and genomic test information in electronic health records: points to consider. Genetics in medicine : official journal of the American College of Medical Genetics. 2008;10(7):495-9.

74. McLaughlin K, Coderre, S. Finding the middle path in tracking former patients in the electronic health record for the purpose of learning. Academic medicine : journal of the Association of American Medical Colleges. 2015;90(8):1007-9.

75. McSherry B. Ethical issues in HealthConnect's shared electronic health record system. Journal of law and medicine. 2004;12(1):60-8.

76. McSherry B. Third party access to shared electronic mental health records: Ethical issues. Psychiatry, Psychology and Law. 2004;11(1):53-62.

77. Meslin EM, Alpert, S. A., Carroll, A. E., Odell, J. D., Tierney, W. M., Schwartz, P. H. Giving patients granular control of personal health information: using an ethics 'Points to Consider' to inform informatics system designers. International journal of medical informatics. 2013;82(12):1136-43.

78. Meslin EM, Schwartz PH. How Bioethics Principles Can Aid Design of Electronic Health Records to Accommodate Patient Granular Control. Journal of general internal medicine. 2015;30(1):3-6.

79. Milton CL. Information sharing: Transparency, Nursing Ethics, and practice implications with electronic medical records. Nursing science quarterly. 2009;22(3):214-9.

80. Neame RLB. Privacy protection for personal health information and shared care records. Informatics in Primary Care. 2014;21(2):84-91.

81. Nielsen BA. Confidentiality and electronic health records: Keeping up with advances in technology and expectations for access. Clinical Practice in Pediatric Psychology. 2015;3(2):175-8.

82. Nielsen BA, Baum RA, Soares NS. Navigating ethical issues with electronic health records in developmental-behavioral pediatric practice. Journal of Developmental and Behavioral Pediatrics. 2013;34(1):45-51.

83. Okada M, Yamamoto, K., Watanabe, K. Conceptual model of health information ethics as a basis for computer-based instructions for electronic patient record systems. Studies in health technology and informatics. 2007;129(Pt 2):1442-6.

84. Ow Yong LM, Tan AWL, Loo CLK, Lim ELP. Risk Mitigation of Shared Electronic Records System in Campus Institutions: Medical Social Work Practice in Singapore. Social Work in Health Care. 2014;53(9):834-44.

85. Phillips W, Fleming DA. Moral and prudential considerations in adopting electronic medical records. Missouri medicine. 2010;107(4):234-9.

86. Phillips W, Fleming, D. Ethical concerns in the use of electronic medical records. Missouri medicine. 2009;106(5):328-33.

87. Pirnejad H, Bal, R., Stoop, A. P., Berg, M. Inter-organisational communication networks in healthcare: centralised versus decentralised approaches. International journal of integrated care. 2007;7:e14.

88. Van der Ploeg I. Positioning the Patient: Normative Analysis of Electronic Patient Records. Methods of information in medicine. 2003;42(4):477-81.

89. Quantin C, Coatrieux G, Allaert FA, Fassa M, Bourquard K, Boire JY, et al. New advanced technologies to provide decentralised and secure access to medical records: Case studies in oncology. Cancer Informatics. 2009;7:217-29.

90. Amarasingham R, Audet, A. M., Bates, D. W., Glenn Cohen, I., Entwistle, M., Escobar, G. J., Liu, V., Etheredge, L., Lo, B., Ohno-Machado, L., Ram, S., Saria, S., Schilling, L. M., Shahi, A., Stewart, W. F., Steyerberg, E. W., Xie, B. Consensus Statement on Electronic Health Predictive Analytics: A Guiding Framework to Address Challenges. EGEMS (Washington, DC). 2016;4(1):1163.

91. Rentmeester C. Heeding humanity in an age of electronic health records: Heidegger, Levinas, and Healthcare. Nursing Philosophy. 2018;19(3).

92. Rigby M, Draper R, Hamilton I. Finding ethical principles and practical guidelines for the controlled flow of patient data. Methods of information in medicine. 1999;38(4-5):345-9.

93. Robertson MD, Kerridge IH. "Through a glass, darkly": The clinical and ethical implications of Munchausen syndrome. Medical Journal of Australia. 2009;191(4):217-9.

94. Rothstein MA. The hippocratic bargain and health information technology. Journal of Law, Medicine and Ethics. 2010;38(1):7-13.

95. Rothstein MA, Talbott MK. Compelled authorizations for disclosure of health records: Magnitude and implications. American Journal of Bioethics. 2007;7(3):38-45.

96. Sade RM. Breaches of health information: are electronic records different from paper records? The Journal of clinical ethics. 2010;21(1):39-41.

97. Samsuri S, Ismail Z, Ahmad R. Adopting a knowledge management concept in securing the privacy of electronic medical record systems. Advances in Intelligent Systems and Computing2013. p. 547-58.

98. Sanelli-Russo S, Folkers, K. M., Sakolsky, W., Fins, J. J., Dubler, N. N. Meaningful Use of Electronic Health Records for Quality Assessment and Review of Clinical Ethics Consultation. The Journal of clinical ethics. 2018;29(1):52-61.

99. Satkoske VB, Parker, L. S. Practicing preventive ethics, protecting patients: challenges of the electronic health record. The Journal of clinical ethics. 2010;21(1):36-8.

100. Scott RE, Jennett P, Yeo M. Access and authorisation in a Glocal e-Health Policy context. International journal of medical informatics. 2004;73(3):259-66.

101. Shenoy A, Appel JM. Safeguarding confidentiality in electronic health records. Cambridge Quarterly of Healthcare Ethics. 2017;26(2):337-41.

102. Shoenbill K, Fost N, Tachinardi U, Mendonca EA. Genetic data and electronic health records: A discussion of ethical, logistical and technological considerations. Journal of the American Medical Informatics Association. 2014;21(1):171-80.

103. Sittig DF, Singh, H. Legal, ethical, and financial dilemmas in electronic health record adoption and use. Pediatrics. 2011;127(4):e1042-7.

104. Slabbert MN. Parental access to minors' health records in the South African health care context: Concerns and recommendations. Medicine and Law. 2005;24(4):743-59.

105. Smolyansky BH, Stark LJ, Pendley JS, Robins PM, Price K. Confidentiality and electronic medical records for behavioral health records: The experience of pediatric psychologists at four children's hospitals. Clinical Practice in Pediatric Psychology. 2013;1(1):18-27.

106. Spector-Bagdady K, Shuman, A. G. Reg-ent within the Learning Health System. Otolaryngology--head and neck surgery : official journal of American Academy of Otolaryngology-Head and Neck Surgery. 2018;158(3):405-6.

107. Spencer A, Low D. The challenge of the information culture for the paediatrician. Archives of Disease in Childhood. 2011;96(12):1167-72.

108. Spriggs M, Arnold, M. V., Pearce, C. M., Fry, C. Ethical questions must be considered for electronic health records. Journal of medical ethics. 2012;38(9):535-9.

109. Stahl BC, Doherty NF, Shaw M, Janicke H. Critical Theory as an Approach to the Ethics of Information Security. Science and Engineering Ethics. 2014;20(3):675-99.

110. Stein HF. Interfaces between electronic medical record (EMR/EHR) technology and people in American medicine: insight. imagination, and relationships in clinical practice. The Journal of the Oklahoma State Medical Association. 2012;105(8):316-9.

111. Strain J, Botin L. A phenomenological perspective on clinical communication and interaction: The case of electronic health records. Journal of Information, Communication and Ethics in Society. 2007;5(1):20-32.

112. Sulmasy LS, Lopez, A. M., Horwitch, C. A. Ethical Implications of the Electronic Health Record: In the Service of the Patient. Journal of general internal medicine. 2017;32(8):935-9.

113. Tehrani N. How Digital Health Technology Aids Physicians. Int J Biomed. 2015;5(2):104-+.

114. Tussey CM, Marcopulos BA, Bush SS. Evolving Roles, Innovative Practice, and Rapid Technology Growth: Remaining Ethical in Modern Clinical Neuropsychology. Psychological Injury and Law. 2015;8(4):281-8.

115. Veronesi JF. Ethical issues in computerized medical records. Critical Care Nursing Quarterly. 1999;22(3):75-80.

116. Wallace IM. Is patient confidentiality compromised with the electronic health record?: a position paper. Computers, informatics, nursing : CIN. 2015;33(2):58-62; quiz E1.

117. Wangenheim PM. Scribes, Electronic Health Records, and the Expectation of Confidentiality. The Journal of clinical ethics. 2018;29(3):240-3.

118. Weis JM, Levy PC. Copy, paste, and cloned notes in electronic health records;prevalence, benefi ts, risks, and best practice recommendations. Chest. 2014;145(3):632-8.

119. Whitehouse D, Duquenoy P. eHealth and ethics: Theory, teaching, and practice. Information and Communication Technologies, Society and Human Beings: Theory and Framework2010. p. 454-65.

120. Wilburn A. Nursing Informatics: Ethical Considerations for Adopting Electronic Records. NASN school nurse (Print). 2018;33(3):150-3.

121. Williams RL, Taylor JF. Four steps to preserving adolescent confidentiality in an electronic health environment. Current Opinion in Obstetrics and Gynecology. 2016;28(5):393-8.

122. Williams H, Spencer, K., Sanders, C., Lund, D., Whitley, E. A., Kaye, J., Dixon, W. G. Dynamic consent: a possible solution to improve patient confidence and trust in how electronic patient records are used in medical research. JMIR medical informatics. 2015;3(1):e3.

123. Wynia M, Dunn K. Dreams and nightmares: Practical and ethical issues for patients and physicians using personal health records. Journal of Law, Medicine and Ethics. 2010;38(1):64-73.
