# Supplementary material for: Examination and diagnosis of electronic patient records and their associated ethics: a scoping literature review
Source: BMC Med Ethics. 2020 Aug 24;21:76. doi: 10.1186/s12910-020-00514-1 (PMC7446190; doi:10.1186/s12910-020-00514-1)
Supplement: Supplementary file 3 — Additional file 3. Appendix C: Thesaurus file. Appendix D: Overview of terms. [file 12910_2020_514_MOESM3_ESM.docx]

# **Appendix C: Thesaurus file**

The VOSviewer thesaurus file means that different variants of the same term are counted as a single term as VOSviewer does not account for synonyms without a thesaurus file specifying them. For example, a variety of terms is used to describe ‘clinician patient relationship’ (e.g. ‘physician patient relationship’; ‘doctor patient relationship’ etcetera). VOSviewer counts these as distinct terms. As a result, a term may not reach the selected threshold of being mentioned in six different articles (which means that term is used in at least ~5% of articles). To address this issue, an approach was chosen to identify all terms (so without the threshold that a term had to appear in at least six articles). From the generated list with all terms, we created this thesaurus file with the term how it appears in an article and right the term which is it counted towards.

Below shows the terms in the thesaurus file. Left shows how the term appears in an article and right the term it should be counted towards.

| **label** | **Count as** |
| --- | --- |
| biase | bias |
| measurement bias | bias |
| possible biase | bias |
| selection bias | bias |
| skilled mental health clinician | clinician |
| dbp clinician | clinician |
| multiple clinician | clinician |
| clinical physician | physician |
| conscientious physician | physician |
| local physician | physician |
| medieval islamic physician | physician |
| clinician patient relationship | clinician patient relationship |
| doctor patient relationship | clinician patient relationship |
| doctorpatient relationship | clinician patient relationship |
| patientphysician relationship | clinician patient relationship |
| physician patient relationship | clinician patient relationship |
| provider patient relationship | clinician patient relationship |
| longer patient physician interaction | clinician patient relationship |
| nursepatient interaction | clinician patient relationship |
| patient doctor interaction | clinician patient relationship |
| doctor | physician |
| medical student | student |
| assigning responsibility | responsibility |
| clinicians responsibility | responsibility |
| collective responsibility | responsibility |
| new responsibility | responsibility |
| patient responsibility | responsibility |
| physicians professional ethical responsibility | responsibility |
| responsibility | responsibility |
| ultimate responsibility | responsibility |
| big data benefit | benefit |
| clinical benefit | benefit |
| immeasurable benefit | benefit |
| long lasting benefit | benefit |
| new benefit | benefit |
| numerous benefit | benefit |
| possible benefit | benefit |
| potential benefit | benefit |
| potential ethical benefit | benefit |
| public health benefit | benefit |
| risk benefit approach | benefit |
| significant benefit | benefit |
| societal benefit | benefit |
| strong benefit | benefit |
| wider public benefit | benefit |
| breaching patient privacy | privacy |
| data privacy | privacy |
| data privacy protection | privacy |
| enhancing privacy | privacy |
| geographical privacy access continuum framework | privacy |
| individual patient privacy | privacy |
| information privacy | privacy |
| medical records privacy | privacy |
| patient centric privacy | privacy |
| patient privacy | privacy |
| patient privacy consideration | privacy |
| patients privacy | privacy |
| personal information privacy | privacy |
| personal medical information privacy | privacy |
| phr portal system level privacy mechanism | privacy |
| poor privacy specific interface design | privacy |
| privacy breach | privacy |
| privacy breech risk | privacy |
| privacy concern | privacy |
| privacy heuristic | privacy |
| privacy issue | privacy |
| privacy law | privacy |
| privacy management solution | privacy |
| privacy preservation | privacy |
| privacy preservation framework | privacy |
| privacy protection | privacy |
| privacy requirement | privacy |
| privacy safeguard | privacy |
| privacy specific heuristic | privacy |
| privacy statement | privacy |
| versus privacy | privacy |
| adolescent confidentiality | confidentiality |
| broader confidentiality protection | confidentiality |
| compromised patientsconfidentiality | confidentiality |
| confidentiality | confidentiality |
| confidentiality concern | confidentiality |
| confidentiality requirement | confidentiality |
| ehr confidentiality specific | confidentiality |
| medical confidentiality | confidentiality |
| patient confidentiality | confidentiality |
| patients confidentiality | confidentiality |
| safeguarding confidentiality | confidentiality |
| breaching patient | privacy |
| data breach | breach |
| medical information breach | breach |
| numerous data breach | breach |
| security breach | breach |
| spectacular breach | breach |
| balancing security sharing | security |
| data security | security |
| electronic data insecurity | security |
| improved security | security |
| information security | security |
| potential security | security |
| pressing security concern | security |
| related security implication | security |
| security awareness mindset | security |
| security breach | security |
| security concern | security |
| security culture | security |
| security issue | security |
| security policy | security |
| security requirement | security |
| security right | security |
| security sharing | security |
| security threat | security |
| certain risk | risk |
| multiple risk | risk |
| multiple specific risk | risk |
| patient care risk | risk |
| patient risk | risk |
| possible risk | risk |
| potential genetic risk | risk |
| potential risk | risk |
| privacy breech risk | risk |
| relative risk | risk |
| risk benefit approach | risk |
| risk management legal consideration | risk |
| risk mitigation | risk |
| risk selection | risk |
| serious risk | risk |
| appropriate informed consent | consent |
| dynamic consent | consent |
| dynamic consent model | consent |
| individual patient consent | consent |
| informed consent | consent |
| noncoerced consent | consent |
| patient consent | consent |
| signed consent | consent |
| specific consent | consent |
| tacit consent | consent |
| access control | control |
| accommodate patient granular control | control |
| adolescent control | control |
| control mechanism | control |
| control should patients have | control |
| ehr access control | control |
| full control | control |
| granular control | control |
| legal control | control |
| patient control | control |
| patient granular control | control |
| patients granular control | control |
| personal control | control |
| data protection | protection |
| information protection | protection |
| legal protection | protection |
| persistent protection | protection |
| privacy protection | protection |
| protection requirement | protection |
| high quality care | quality |
| lower quality | quality |
| quality | quality |
| quality assessment | quality |
| quality control procedure | quality |
| quality framework | quality |
| quality healthcare service | quality |
| quality improvement | quality |
| quality improvement safety initiative | quality |
| quality pitfall | quality |
| quality problem | quality |
| office efficiency | efficiency |
| patient autonomy | autonomy |
| patient safety | safety |
| patient safety implication | safety |
| potential ehr safety issue | safety |
| quality improvement safety initiative | safety |
| adequate transparency | transparency |
| full transparency | transparency |
| health information transparency | transparency |
| scientific transparency | transparency |
| testingtransparency | transparency |
| mutual trust | trust |
| patient trust | trust |
| public trust | trust |
| trust management | trust |
| trust management environment | trust |
| trustworthy ehr solution | trust |
| way trust | trust |
| data integrity | integrity |
| somatising patient | patient |
| subsequent patient | patient |
| individual patient | patient |
| former patient | patient |
| complex patient | patient |
| adolescent patient | patient |
| health care professional | healthcare professional |
| health professionals | healthcare professional |
| health information professionals | health information professional |
| software personnel | software personnel |
| health professional | healthcare professional |
| healthcare professional | healthcare professional |
| ict professional | ict professional |
| informatics professional | ict professional |
| major teaching hospital | hospital |
| singapore general hospital | hospital |
| urban hospital | hospital |
| danish hospital system | hospital system |
| hospital management system | hospital system |
| hospital sector | hospital system |
| hospital system | hospital system |
| busy health system | health system |
| health care delivery system | health system |
| health care system | health system |
| healthcare system | health system |
| modern health care system | health system |
| nationwide system | health system |
| us health system | health system |
| modern health care | health system |
| healthcare service | health system |
| health care service | health system |
| modern health care system | health system |
| health care provider | healthcare provider |
| care provider | healthcare provider |
| dutch healthcare provider | healthcare provider |
| appropriate care provider | healthcare provider |
| behavioral health provider | healthcare provider |
| provider patient relationship | healthcare provider |
| healthcare organization | healthcare provider |
| international organization | organization |
| non governmental organization | organization |
| organization | organization |
| high user | user |
| human user | user |
| individual end user | user |
| third party user | user |
| civil society | society |
| adolescent care | care |
| cancer care | care |
| clinical care | care |
| direct care | care |
| direct patient care | care |
| confidential care | care |
| effective clinical care | care |
| effective health care | care |
| efficacious care | care |
| health care | care |
| healthcare | care |
| individual care | care |
| medical care | care |
| optimal patient care | care |
| overall patient care | care |
| patient care | care |
| primary care | care |
| social care data | care |
| reproductive health care | care |
| sensitive health care data | care |
| usual care | care |
| adolescent child | child |
| older children | child |
| clinic staff | staff |
| laboratory staff | staff |
| medical student | student |
| medical students track former patients | student |
| medical researcher | researcher |

# **Appendix D: Overview of terms**

The table below presents the full list of terms generated by VOSviewer. We have selected 16 terms with strong ethical connotations from this lists to be discussed in the body of the paper. We did not select ambiguous terms, such as ‘access’ or ‘ability’ which may or may not have ethical relevance depending on the context in which they are used. Because of this method, we may have missed terms that did have ethical relevance across multiple articles. However, our aim was not to identify all ethical principles and values but discuss some of the most frequently mentioned ones to identify factors influencing an ethical assessment.

| \| id \| label \| Occurrences \| \| --- \| --- \| --- \| \| 1 \| ability \| 6 \| \| 2 \| access \| 36 \| \| 3 \| addition \| 6 \| \| 4 \| adoption \| 11 \| \| 5 \| advance \| 10 \| \| 6 \| age \| 7 \| \| 7 \| application \| 11 \| \| 8 \| approach \| 20 \| \| 9 \| article \| 30 \| \| 10 \| attention \| 12 \| \| 11 \| author \| 11 \| \| 12 \| autonomy \| 9 \| \| 13 \| basis \| 9 \| \| 14 \| benefit \| 26 \| \| 15 \| breach \| 12 \| \| 16 \| care \| 52 \| \| 17 \| challenge \| 24 \| \| 18 \| change \| 10 \| \| 19 \| child \| 6 \| \| 20 \| clinician \| 14 \| \| 21 \| clinician patient relationship \| 13 \| \| 22 \| concept \| 6 \| \| 23 \| concern \| 20 \| \| 24 \| confidentiality \| 28 \| \| 25 \| consent \| 21 \| \| 26 \| context \| 11 \| \| 27 \| control \| 11 \| \| 28 \| cost \| 9 \| \| 29 \| country \| 6 \| \| 30 \| data \| 50 \| \| 31 \| demand \| 6 \| \| 32 \| development \| 19 \| \| 33 \| documentation \| 11 \| \| 34 \| efficiency \| 7 \| \| 35 \| ehr \| 44 \| \| 36 \| electronic health record \| 68 \| \| 37 \| electronic medical record \| 27 \| \| 38 \| electronic patient record \| 13 \| \| 39 \| electronic record \| 7 \| \| 40 \| emr \| 13 \| \| 41 \| ethic \| 26 \| \| 42 \| ethical challenge \| 7 \| \| 43 \| ethical concern \| 11 \| \| 44 \| ethical consideration \| 7 \| \| 45 \| ethical implication \| 10 \| \| 46 \| ethical issue \| 22 \| \| 47 \| ethical principle \| 8 \| \| 48 \| ethics \| 12 \| \| 49 \| example \| 12 \| \| 50 \| experience \| 12 \| \| 51 \| expert \| 6 \| \| 52 \| factor \| 8 \| \| 53 \| framework \| 11 \| \| 54 \| goal \| 6 \| \| 55 \| health \| 26 \| \| 56 \| health data \| 11 \| \| 57 \| health information \| 22 \| \| 58 \| health record \| 15 \| \| 59 \| health system \| 18 \| \| 60 \| healthcare professional \| 6 \| \| 61 \| healthcare provider \| 12 \| \| 62 \| hospital \| 12 \| \| 63 \| implementation \| 17 \| \| 64 \| implication \| 13 \| \| 65 \| importance \| 10 \| \| 66 \| information \| 45 \| \| 67 \| institution \| 14 \| \| 68 \| issue \| 36 \| \| 69 \| law \| 8 \| \| 70 \| medical record \| 20 \| \| 71 \| model \| 13 \| \| 72 \| need \| 12 \| \| 73 \| opportunity \| 10 \| \| 74 \| paper \| 34 \| \| 75 \| patient \| 63 \| \| 76 \| person \| 10 \| \| 77 \| physician \| 19 \| \| 78 \| policy \| 18 \| \| 79 \| practice \| 23 \| \| 80 \| principle \| 17 \| \| 81 \| privacy \| 41 \| \| 82 \| problem \| 16 \| \| 83 \| process \| 14 \| \| 84 \| protection \| 10 \| \| 85 \| provider \| 7 \| \| 86 \| quality \| 18 \| \| 87 \| question \| 24 \| \| 88 \| recommendation \| 9 \| \| 89 \| record \| 24 \| \| 90 \| requirement \| 10 \| \| 91 \| research \| 33 \| \| 92 \| responsibility \| 9 \| \| 93 \| review \| 11 \| \| 94 \| right \| 7 \| \| 95 \| risk \| 22 \| \| 96 \| role \| 12 \| \| 97 \| safety \| 10 \| \| 98 \| security \| 26 \| \| 99 \| society \| 8 \| \| 100 \| student \| 6 \| \| 101 \| study \| 13 \| \| 102 \| system \| 42 \| \| 103 \| technology \| 31 \| \| 104 \| time \| 14 \| \| 105 \| training \| 8 \| \| 106 \| transparency \| 7 \| \| 107 \| treatment \| 11 \| \| 108 \| trust \| 6 \| \| 109 \| type \| 9 \| \| 110 \| united states \| 6 \| \| 111 \| use \| 52 \| \| 112 \| value \| 11 \| \| 113 \| way \| 21 \| |  |  |
| --- | --- | --- | --- | --- | --- | --- | --- | --- | --- | --- | --- | --- | --- | --- | --- | --- | --- | --- | --- | --- | --- | --- | --- | --- | --- | --- | --- | --- | --- | --- | --- | --- | --- | --- | --- | --- | --- | --- | --- | --- | --- | --- | --- | --- | --- | --- | --- | --- | --- | --- | --- | --- | --- | --- | --- | --- | --- | --- | --- | --- | --- | --- | --- | --- | --- | --- | --- | --- | --- | --- | --- | --- | --- | --- | --- | --- | --- | --- | --- | --- | --- | --- | --- | --- | --- | --- | --- | --- | --- | --- | --- | --- | --- | --- | --- | --- | --- | --- | --- | --- | --- | --- | --- | --- | --- | --- | --- | --- | --- | --- | --- | --- | --- | --- | --- | --- | --- | --- | --- | --- | --- | --- | --- | --- | --- | --- | --- | --- | --- | --- | --- | --- | --- | --- | --- | --- | --- | --- | --- | --- | --- | --- | --- | --- | --- | --- | --- | --- | --- | --- | --- | --- | --- | --- | --- | --- | --- | --- | --- | --- | --- | --- | --- | --- | --- | --- | --- | --- | --- | --- | --- | --- | --- | --- | --- | --- | --- | --- | --- | --- | --- | --- | --- | --- | --- | --- | --- | --- | --- | --- | --- | --- | --- | --- | --- | --- | --- | --- | --- | --- | --- | --- | --- | --- | --- | --- | --- | --- | --- | --- | --- | --- | --- | --- | --- | --- | --- | --- | --- | --- | --- | --- | --- | --- | --- | --- | --- | --- | --- | --- | --- | --- | --- | --- | --- | --- | --- | --- | --- | --- | --- | --- | --- | --- | --- | --- | --- | --- | --- | --- | --- | --- | --- | --- | --- | --- | --- | --- | --- | --- | --- | --- | --- | --- | --- | --- | --- | --- | --- | --- | --- | --- | --- | --- | --- | --- | --- | --- | --- | --- | --- | --- | --- | --- | --- | --- | --- | --- | --- | --- | --- | --- | --- | --- | --- | --- | --- | --- | --- | --- | --- | --- | --- | --- | --- | --- | --- | --- | --- | --- | --- | --- | --- | --- | --- | --- | --- | --- | --- | --- | --- | --- | --- | --- | --- | --- | --- | --- | --- | --- | --- | --- | --- | --- | --- | --- | --- | --- | --- | --- | --- | --- | --- | --- |
